# Electronic Structure of the Au Nanoparticle-TiO_2_ Heterojunction: Influence of Nanoparticle Size, Shape, Oxygen Vacancies, and Temperature

**DOI:** 10.1021/acs.jpcc.5c05449

**Published:** 2025-12-06

**Authors:** Carlos Mora Perez, Drew M. Glenna, Ernest Hermosillo, Zachery Donnellan, Soumyadeep Ghosh, Oliver Gessner, Jin Qian

**Affiliations:** † Chemical Sciences Division, 1666 Lawrence Berkeley National Laboratory, Berkeley, California 94720, United States; ‡ Department of Nuclear Engineering & Industrial Management, 5640 University of Idaho, Idaho Falls, Idaho 83402, United States; § Department of Chemistry, University of California, Berkeley, California 94720, United States

## Abstract

The electronic structure of the gold nanoparticle–titanium dioxide (AuNP–TiO_2_) heterojunction plays a critical role for charge transfer and recombination dynamics that underpin its photocatalytic function. However, building a representative model to capture the key physics remains a significant challenge. Here, we investigate the influence of the AuNP size and shape, as well as oxygen vacancy (V_O_) defects at the anatase-phase TiO_2_ (101) surface and the temperature of the heterojunction, on its interfacial electronic properties. Using density functional theory (DFT), we compare the closed-shell Au_20_ and open-shell Au_19_ clusters interfaced with pristine and V_O_ defect TiO_2_ surfaces. We find that the presence of a V_O_ defect transforms pure TiO_2_ from a p-type to an n-type semiconductor, reversing the interfacial band bending from downward to upward. For the heterosystem, density of states (DOS) analysis shows that V_O_ minimally affects the open-shell Au_19_–TiO_2_ system, but it significantly alters the closed-shell Au_20_–TiO_2_ system, converting it from p-type to n-type at the Γ-point at 0 K. Furthermore, ab initio molecular dynamics (AIMD) simulations at 300 K reveal significant thermal fluctuations in AuNP positions relative to the TiO_2_ surface. These fluctuations result in dynamic variations in the gap between the highest occupied molecular orbital (HOMO) and the lowest unoccupied molecular orbital (LUMO) in all systems studied: Au_19_NP*–*Pristine TiO_2_, Au_20_NP*–*Pristine TiO_2_, Au_19_NP*–*V_O_ defect TiO_2_, and Au_20_NP*–*V_O_ defect TiO_2_. Complementary Bader charge analysis performed at both 0 K and finite-temperature AIMD snapshots supports the emergence of an upward band bending and the formation of a Schottky barrier at the V_O_ containing heterojunctions. Notably, we find that at finite temperature, an Au atom can dynamically passivate the V_O_, leading to a pronounced widening of the HOMO–LUMO gap in the AuNP–V_O_ defect TiO_2_ heterojunctions. Our computational findings underscore the pivotal role of the V_O_ defect and thermal effects in modulating interfacial band alignment, electronic states, and HOMO*–*LUMO gaps, providing insights for designing Au–TiO_2_ heterojunctions with tailored electronic properties.

## Graphical abstract

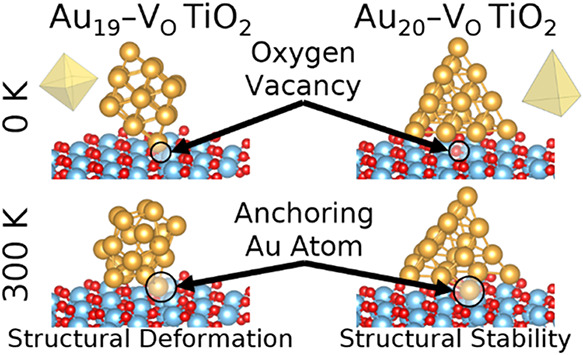

## 1 Introduction

The development of heterogeneous materials for photoconversion processes presents both exciting opportunities and persistent challenges in the field of photocatalysis, particularly for solar-driven reactions such as water splitting and pollutant degradation.^1–3^ Among photocatalytically active materials, TiO_2_ is drawing significant attention due to its low cost and favorable optoelectronic properties.^3–6^ However, its practical application is limited by two key factors: poor visible light absorption due to its wide band gap and rapid recombination of photogenerated charge carriers, both of which reduce its overall photocatalytic efficiency.^3,4,7^ To overcome these limitations, various strategies have been explored, including elemental doping, defect engineering, and the construction of heterojunctions with metal nanoparticles.^3,4,7^ Among these, coupling TiO_2_ with plasmonic metal nanoparticles has emerged as a promising route to enhance charge separation and extend light absorption, thereby improving photocatalytic performance.

In particular, integrating gold nanoparticles (AuNPs) with TiO_2_ has proven to be an effective strategy. The formation of AuNP*–*TiO_2_ heterojunctions offers several key advantages. First, the localized surface plasmon resonance (LSPR) of AuNPs extends the system’s light absorption into the visible range, enabling more efficient utilization of the solar spectrum.^8–10^ Second, the plasmonic excitation of AuNPs generates energetic, nonequilibrium (“hot”) electrons that can be injected into the TiO_2_ conduction band (CB), driving photocatalytic reactions.^11–13^ Third, the metal–semiconductor interface facilitates charge separation and reduces the recombination of photogenerated electron–hole pairs, resulting in prolonged carrier lifetimes that enhance photocatalytic activity.^11,12,14–17^ To fully understand the physics underlying these concepts, their limitations, and possible opportunities for improvement, it is essential to develop carefully constructed models that capture the electronic structure and interfacial interactions governing charge transfer at the AuNP–TiO_2_ interface.

A detailed electronic structure analysis is critical for understanding interfacial charge transfer processes in these systems. One important property is the work function of the individual metal and semiconductor components, which provides insight into the band alignment and the formation of the Schottky barrier at the interface. For bulk gold, experimental measurements report work function values ranging from 5.20 to 5.40 eV, depending on the crystalline facet: Au (111), 5.26 ± 0.04 eV;^18^ Au (111) 5.35 eV;^19^ Au (110), 5.20 ± 0.04 eV;^18^ Au (100), 5.22 ± 0.04 eV;^18^ and polycrystalline 5.22 ± 0.05 eV,^20^ 5.40 ± 0.13 eV.^21^ In contrast, reported values for the work function of anatase TiO_2_ (101) surface vary across a considerably larger range, from 4.40 to 7.54 eV. For example, theoretical estimates range from 6.58 to 7.54 eV (6.58 eV (DFT),^22^ 7.46 eV (DFT+U),^23^ 7.54 (DFT)^23^) and experimental values vary between 4.40 and 6.72 eV, depending on the sample environment (4.40 eV (air),^24^ 4.64 eV (vacuum),^24^ and 4.62–6.72 eV (vacuum, various surface conditions)^25^). This variability highlights the sensitivity of TiO_2_’s electronic properties to surface structure and defect states. In particular, oxygen vacancies on the TiO_2_ surface are known to create localized trap states, which can lower the work function by shifting the Fermi level (*E*_F_).^26,27^ Given the important role these defects play, building models that explicitly account for such surface features is critical.

Beyond the work function, the DOS and the energetic positions of the HOMO, LUMO, and *E*_F_ are essential descriptors of the electronic structure. The magnitude of the HOMO–LUMO gap affects nonadiabatic recombination dynamics of photogenerated charge carriers, with narrower gaps typically associated with higher recombination rates and wider gaps linked to longer carrier lifetimes.^28,29^ Furthermore, analysis of the spatial distribution and chemical character of the HOMO and LUMO wave functions via orbital-resolved charge density plots reveals the nature of these edge states. The position of *E*_F_ relative to the band edges serves as a crucial benchmark for evaluating whether the model system is n-type or p-type. Together, these electronic properties provide crucial insights into how interfacial structure affects photocatalytic activity and inform the rational design of advanced materials. These properties can be systematically evaluated through DFT calculations, which have been instrumental in uncovering the electronic structure and interfacial behavior of AuNP–TiO_2_ heterojunctions.^12,30–38^

In this study, we employ the DFT methodology to analyze the electronic structure of model AuNP-TiO_2_ systems under static (0 K) conditions and under elevated temperatures (300 K). Specifically, we investigate how the nanoparticle shape influences interfacial electronic properties by comparing two similarly sized AuNPs, the open-shell Au_19_ and the closed-shell Au_20_. Both the electronic structure (open-shell vs closed-shell) and the geometrical shape of the nanoparticle play important roles in determining the heterojunction characteristics. These properties are inherently linked through the number of constituent Au atoms, which simultaneously governs symmetry and electronic configuration. The AuNPs are supported on an anatase TiO_2_ (101) surface, known for its high photocatalytic activity.^7,39^ To explore defect-induced effects, we incorporate surface oxygen vacancy (V_O_) and examine its impact on electronic properties. We find that V_O_ is critical for establishing Schottky barriers at the Au–TiO_2_ interface, as they enable interfacial charge separation and upward band bending, in stark contrast to the downward band bending observed in pristine systems. Additionally, we complement the static DFT analysis with AIMD simulations at 300 K to capture finite-temperature effects. These simulations reveal that the Au_19_NP is more flexible and undergoes significant structural distortion, losing its 0 K symmetry at higher temperatures, whereas the Au_20_NP system largely retains its pyramidal geometry. The thermally induced structural changes result in larger average HOMO–LUMO gaps compared to their static 0 K counterparts, with the exception of the Au_20_NP–pristine TiO_2_ heterojunction. Notably, the increase in the HOMO–LUMO gap is more pronounced in the V_O_ defect systems than in the pristine cases. This gap-widening behavior is traced to a dynamic vacancy-filling process, where an Au atom occupies and passivates the V_O_ at finite temperature, highlighting the critical role of thermal effects at the interface. These findings highlight the importance of both model construction and thermal fluctuations in accurately characterizing interfacial electronic properties, offering valuable insights into the design and modeling of next-generation photocatalytic materials.

## 2 Theoretical Methods

### 2.1 Static DFT VASP Parameters

Details regarding the construction of the TiO_2_ slab models, Au nanoparticle models, and Au–TiO_2_ heterojunctions are provided in the Supporting Information (Sections S1–S5). All calculations are performed using the DFT formalism within the Vienna ab initio simulation package (VASP) software.^40,41^ The PBE functional^42^ at the generalized gradient approximation (GGA) level, has been used for all systems. The following projector augmented wave (PAW) pseudopotentials^43,44^ were employed: PAW_PBE Ti 08Apr2002, PAW_PBE O 08Apr2002, and PAW_PBE Au 04Oct2007. For Au, the 5d^10^6s^1^ valence electrons were treated explicitly, and the PAW pseudopotential includes scalar relativistic effects to account for the contraction of the 6s orbital, expansion of the 5d orbitals, and the resulting influence on Au–Au and Au–O bonding.^45,46^ In all static VASP calculations, the energy cutoff for the plane-wave basis set is converged at a threshold of 600 eV (ENCUT = 600), which is 1.5 times larger than the largest cutoff value specified in the pseudopotential files, ensuring complete convergence of the total energy. The electronic self-consistent field (SCF) loop is set to an energy convergence threshold of 10^–8^ eV (EDIFF = 1 × 10^–8^). During geometry optimization, the convergence criteria for all atomic forces are set to <0.01 eV/Å (EDIFFG = −0.01), and the Brillouin zone (BZ) is sampled using a 5 × 5 × 1 Γ-centered k-point grid. When evaluating the static electronic structure properties (DOS, work function, charge densities), the BZ is reduced to the Γ-point to limit computational costs. Additionally, a Hubbard U parameter of 6.9 eV is applied to the Ti 3d states to replicate the experimentally observed bulk TiO_2_ bandgap^47^ for all electronic structure calculations. The PBE+U approach is used as opposed to hybrid functionals such as HSE,^48^ because PBE+U offers an optimal balance between computational feasibility and accuracy for complex Au–TiO_2_ interfaces,^12,49–53^ particularly for the computationally demanding AIMD simulations that are central to our study. While hybrid functionals could provide more quantitatively accurate band gaps, the PBE+U method is well-established to reliably capture the qualitative trends of interest at lower computational cost. If the slab model is asymmetric along the direction normal to the surface (*c*-direction), a dipole correction term is applied using IDIPOL = 3 to remove electrostatic interactions between periodic images. This is necessary for the V_O_ defect TiO_2_ slab and all Au–TiO_2_ heterojunction models, where the asymmetry introduces an intrinsic dipole moment along c. In all cases, VASP automatically positions the dipole correction layer at the midpoint of the vacuum region, far from both the TiO_2_ surface and the Au nanoparticle (when adsorbed to the surface), ensuring no overlap or artificial interaction. The planar-averaged electrostatic potentials for the pristine and oxygen-vacancy slabs (Figure S7.1) confirm that the potential of the vacuum region remains flat, and that the applied correction does not introduce any tilt.

### 2.2 AIMD VASP Parameters

AIMD ground-state simulations are performed using a 1 × 1 × 1 Γ-centered k-point mesh within the VASP software package. The energy cutoff for the plane-wave basis set is set to 400 eV (ENCUT = 400), and a time step of 1 fs (POTIM = 1) is used in conjunction with a Nose-Hoover thermostat. Geometry-optimized structures serve as the starting configurations for the AIMD simulations. The central layer of the TiO_2_ slab is kept fixed throughout the simulations. Initially, the systems are thermally equilibrated under the canonical ensemble (NVT) for 3 ps, with velocities scaled every 10 fs at a temperature of 300 K. This is followed by an 8 ps production run under the microcanonical ensemble (NVE), generating trajectories at 300 K. For further analysis, the last 6 ps segment of the trajectory is selected to ensure that thermal equilibrium is reached.

## 3 Results and Discussion

### 3.1 TiO_2_ Surface Modeling

To study the Au-TiO_2_ heterojunction effectively, it is crucial to understand the individual components that contribute to its overall behavior. First, we explore the anatase TiO_2_ (101) surface, with a focus on the influence of oxygen vacancies on its electronic structure compared to the clean, pristine configuration as shown in Figure 1a. Surface defects strongly influence the electronic properties of anatase TiO_2_.^26,54^ Oxygen vacancies are particularly common surface defects with a low formation energy.^26^ Here, we remove a single neutral oxygen atom (an oxygen atom with six valence electrons) from the center of the topmost TiO_2_ surface (layer 1). Figure 1b shows the location of the V_O_ on the TiO_2_ slab model.

**Figure 1:**
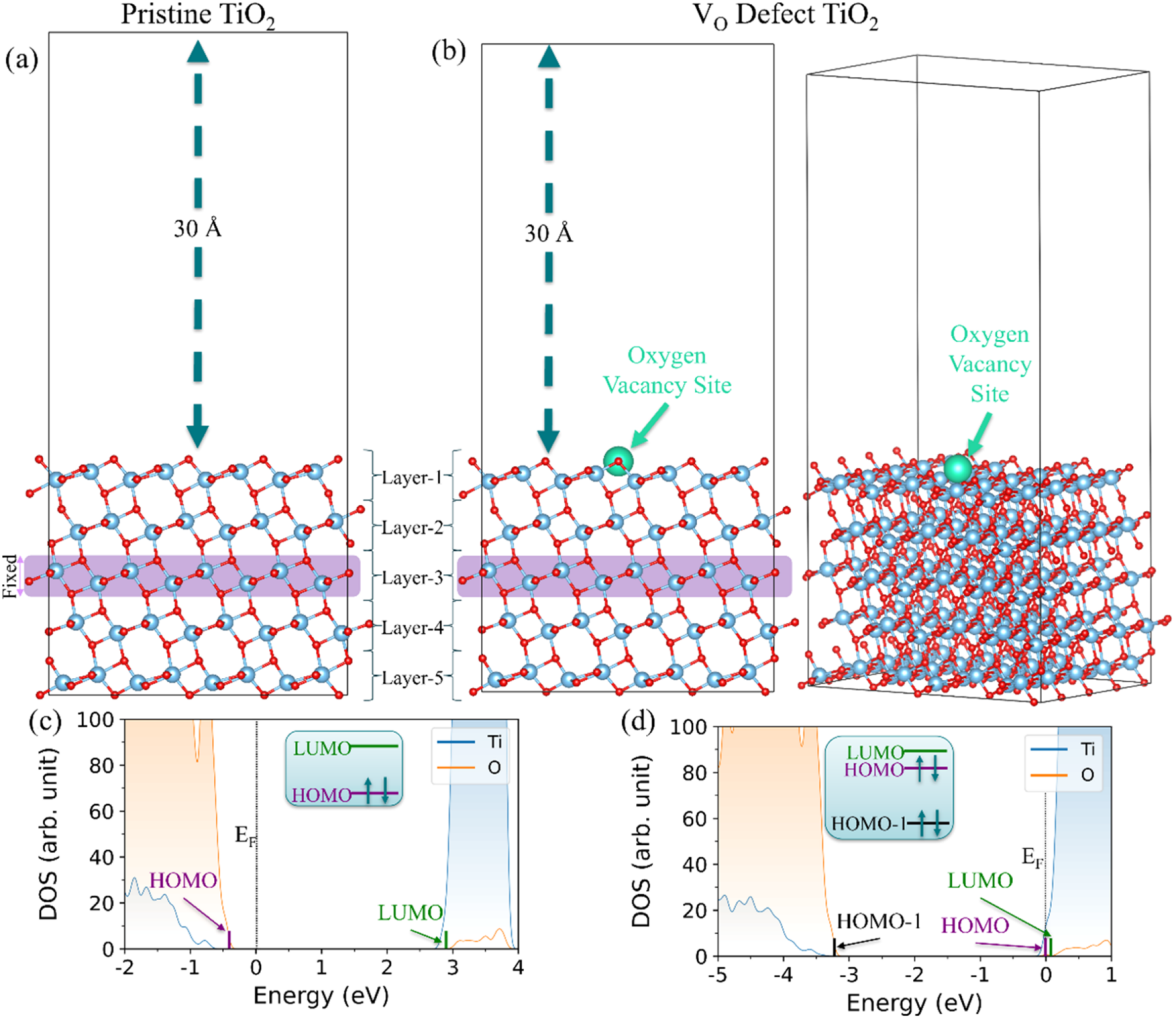
(a) Pristine surface slab model of anatase TiO_2_ (101) using a 5-layer thickness, 2 × 4 surface area (21.0 Å × 15.3 Å), and a 30 Å vacuum region. The atomic positions within a single center layer (layer 3) are fixed in the model, as highlighted in purple. (b) Side and tilted view of the surface slab model with an introduced V_O_ defect. As highlighted by the green circle, a single oxygen atom is removed from the topmost TiO_2_ layer (layer 1). (c, d) DOS at the Γ-point and 0 K of (c) the pristine surface slab model and (d) the V_O_ defect surface slab. The DOS are color-coded by elemental contributions (Ti: blue, O: orange), and *E*_F_ is set to zero. The inserts show the ground state electron occupancies at the HOMO–1 (black), HOMO (purple), and LUMO (green) levels. The structures are optimized at the PBE DFT level at 0 K.

Electronic structure changes induced by the V_O_ defect are evident from a comparison of the DOS plots in Figure 1c,d. In all cases the DOS smearing parameter (σ) is set to 0.05 eV and results in the extension of the DOS curve beyond the calculated eigenvalues shown as sticks. A detailed account of the DOS definition is given in SI Section S4. Although the TiO_2_ systems are modeled using 2D slab geometries, we use molecular nomenclature, such as “HOMO–1”, “HOMO”, and “LUMO”, to describe specific electronic states, depending on their occupancies. Within this nomenclature, the conventional valence band maximum (VBM) and conduction band minimum (CBM) may correspond to different orbitals. For example, while the VBM is typically associated with the HOMO and the CBM with the LUMO for a semiconductor with no gap states, introduction of an occupied shallow gap state may lead to a new HOMO and to the VBM becoming the HOMO–1 state, as illustrated in Figure 1d. To clearly differentiate between electronic states based on their occupancies and the band edge states, we use the terms “HOMO–1”, “HOMO” and “LUMO” in conjunction with “VBM” and “CBM” throughout this work.

The element-specific DOS plot for the pristine TiO_2_ (101) surface (Figure 1c) shows that the HOMO is primarily derived from oxygen states. In contrast, the titanium states dominate the LUMO, consistent with previous findings.^55^ The orbital-resolved charge density plots for the HOMO and the LUMO states further reveal that the band edge states are delocalized across all 5 TiO_2_ layers (Figure S6.1a). These states are separated by a HOMO–LUMO gap of 3.34 eV for the pristine surface slab model. Although anatase TiO_2_ is an intrinsic n-type semiconductor,^56^ our pristine surface model exhibits p-type behavior with *E*_F_ located energetically near the VB states. The definition of *E*_F_ within the VASP calculations is described in more detail in SI Section S7.

Introducing a V_O_ defect in the topmost TiO_2_ surface layer (layer 1) creates a localized charge trap that significantly impacts the electronic properties of the TiO_2_ surface. The trap state is filled and shown as the HOMO level in the DOS (Figure 1d), shifting *E*_F_ closer to the CB (and further away from VB states), and restoring the expected n-type semiconductor character. The HOMO is highly localized near the V_O_ site on the V_O_ defect TiO_2_ model (Figure S6.1b), supporting its characterization as a trap state. In contrast, the HOMO–1 and LUMO states are delocalized across all 5 and the top 4 TiO_2_ layers, respectively, (Figure S6.1b), reminiscent of the HOMO and LUMO states of the pristine surface slab (Figure S6.1a).

Further analysis of the V_O_ defect TiO_2_ DOS (Figure 1d) reveals that the formation of the trap state introduces two distinct energy gaps. The first is the HOMO–1–LUMO gap (3.40 eV), which is slightly larger than the pristine HOMO–LUMO gap (3.34 eV). The second, and more critical gap is the HOMO–LUMO gap, which narrows significantly to 0.08 eV. This reduction results directly from the V_O_ induced trap state, which becomes the new HOMO. Defect induced trap states have been linked to changes in charge carrier lifetimes, significantly impacting photophysical and photochemical performance.^57–60^ However, an in-depth exploration of how this particular trap state influences carrier lifetime lies beyond the scope of the current study.

We perform a Bader charge analysis to assess the charge (re)­distribution at the interface. The reported Bader charge of an atom is the valence electron count minus the total Bader population associated with each atom. We first examine the pristine and V_O_ defect TiO_2_ slabs separately. This charge distribution is reported by layer-resolved Bader charge analysis (Table S12.1). In the pristine TiO_2_ surface slab model, the charge distribution is relatively uniform across all five layers. The surface layers exposed to vacuum (layers 1 and 5) exhibit slight positive charge accumulation (+0.18e and +0.19e, respectively) due to structural relaxation, while the internal layers (layers 2–4) show minor negative charge buildup (−0.08e, −0.16e, and −0.13e, respectively), with the central layer 3 (with atomic positions fixed to bulk parameters) holding the highest negative charge (−0.16e). Introducing a V_O_ significantly disrupts this charge distribution, causing pronounced charge localization: the layer containing the V_O_ (layer 1) accumulates a considerably larger positive charge (+0.56e), while layers 2 and 3 become more negatively charged (−0.34e, −0.25e, respectively) compared to the pristine slab, offsetting the accumulating positive charge at the vacancy site.

### 3.2 Band Bending at the Au–TiO_2_ Heterojunction

To understand the electronic structure of the Au–TiO_2_ heterojunction, we begin by examining one of its most fundamental interfacial properties, the direction of the band bending. We can estimate the band bending behavior (upward vs downward) within the Schottky model by examining the work function difference (ΔΦ = Φ_M_ – Φ_S_) between the metal (Φ_M_) and the semiconductor (Φ_S_). Here, the sign of ΔΦ provides meaningful information: a positive value indicates upward band bending, whereas a negative value indicates downward band bending at the metal–semiconductor interface. We note, however, that the magnitude of ΔΦ carries less significance, as it does not directly correspond to the barrier height at the interface. In the upward band bending picture, the Schottky barrier height (Φ_SB_) of a metal–semiconductor heterojunction can be estimated by the difference between the work function of the metal (Φ_M_) and the electron affinity of the semiconductor (χ_S_); Φ_SB_=Φ_M_ – χs.^61,62^ The determination of this quantity, however, is beyond the scope of the current work as we cannot accurately calculate electron affinities at the interface within VASP. The formation of a Schottky barrier through upward band bending has often been employed to rationalize the decreased probability of the injected electrons in the semiconductor to recombine with holes in the metal and a corresponding increase in carrier lifetimes.^63–65^

The work function, Φ, is calculated as the difference between the vacuum potential (ϕ_vacuum_) and *E*_F_.

1 $$\Phi =\phi_{\mathrm{vacuum}}-E_{F}$$

Here, ϕ_vacuum_ is defined as the electrostatic potential sufficiently far from the surface, where an electron placed at this position experiences no influence from the surface. Therefore, accurate determination of ϕ_vacuum_ relies on including a sufficiently large vacuum region to ensure convergence. Numerically, ϕ_vacuum_ corresponds to the flat region of the planar-averaged electrostatic potential along the c-direction. The planar-averaged electrostatic potential along the *c*-direction at 0 K for pristine and V_O_ defect TiO_2_ is shown in Figure S7.1 to clarify the calculation of the work function.

The calculated work function of the pristine TiO_2_ surface at the Γ-point and 0 K is 6.80 eV, which aligns with the upper limit of experimentally observed values (see Section 1). Meanwhile, the work function of the V_O_ defect TiO_2_ surface is significantly lower, at 4.02 eV–a 41% reduction compared to the pristine surface. This decrease suggests that oxygen vacancies may account for some of the variability reported in experimental work function values, ranging from 4.4 to 6.7 eV.^24,25^ Figure 2 illustrates the relative magnitudes of Φ_S_ and Φ_M_ at the interface between a Au (111) surface and (a) the pristine and (b) the V_O_ defect TiO_2_ (101) surface. This alignment leads to distinct band bending behaviors, which directly affect electron transfer across the interface. Within a simple Schottky model,^66^ the larger work function of the pristine TiO_2_ (101) surface (Φ_S_ = 6.80 eV) compared to Au (111) (Φ_M_ = 5.03 eV), results in a downward band bending at the interface (Figure 2a). The negative work function difference (ΔΦ = −1.77 eV, Φ_M_ < Φ_S_) indicates that no Schottky barrier is formed. This scenario is generally unfavorable for photocatalytic applications relying on electron injection from AuNPs into the TiO_2_ substrate. Injected electrons can easily return to the AuNP, recombine with photogenerated holes, and consequently reduce the likelihood of harvesting hot carriers for chemical reactions.

**Figure 2:**
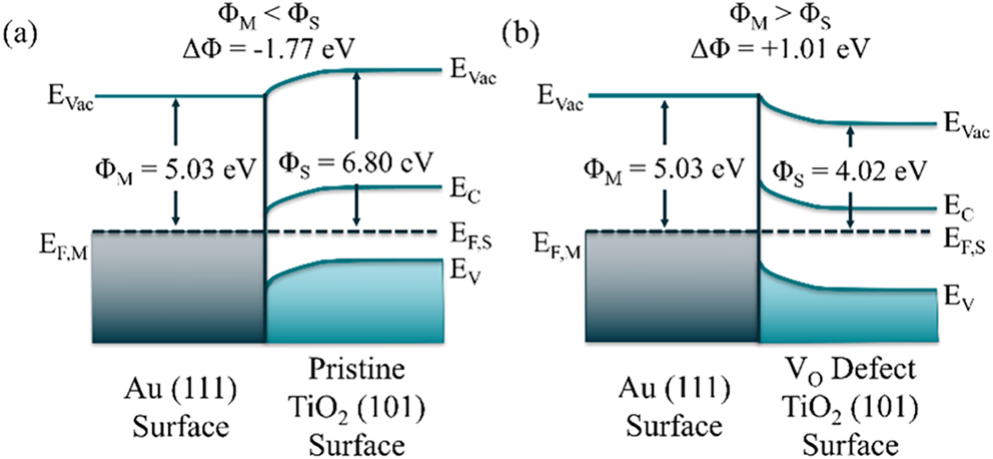
Qualitative estimate of the band bending behavior at the Au–TiO_2_ heterojunction at the Γ-point at 0 K. (a) The work function of the Au (111) surface (Φ_M_ = 5.03 eV) is smaller than the work function of the pristine TiO_2_ (101) surface (Φ_S_ = 6.80 eV), resulting in a “downward” band bending as Φ_M_ < Φ_S_. (b) The work function of the Au (111) surface (Φ_M_ = 5.03 eV) is larger than the work function of the V_O_ defect TiO_2_ (101) surface (Φ_S_ = 4.02 eV). The oxygen defect shifts *E*_F_ from near the VBM to near the CBM, thereby lowering the work function for TiO_2_. In this case, the heterojunction alignment is marked by an “upward” band bending as Φ_M_ > Φ_S_.

In contrast, introducing a V_O_ on the TiO_2_ surface forms a heterojunction with the TiO_2_ (101) surface characterized by upward band bending (Φ_M_ > Φ_S_), with ΔΦ = +1.01 eV as shown in Figure 2b. This effect arises because the V_O_ defect generates a localized trap state near the surface *E*_F_, shifting it closer to the CBM, as discussed in Section 3.1. The positive ΔΦ results in the generation of a Schottky barrier that inhibits electron back transfer to Au, enhancing carrier lifetimes.^67,68^

The qualitative differences between the scenarios in Figure 2a,b highlight the importance of careful defect engineering when designing Au–TiO_2_ heterojunctions for optimized device performance. Notably, AuNPs reproduce similar qualitative trends as those discussed with Au (111) surface in Figure 2. We chose Au_20_ for its well-documented stability, experimental synthesizability, tetrahedral shape, and closed-shell configuration.^69–71^ To evaluate the impact of geometry, size, and electronic structure effects, we also examine a similarly sized Au_19_ cluster, created via the Wulff construction method.^72^ Au_19_ possesses an octahedral geometry and open-shell nature, giving it a distinct electronic structure despite its comparable size to Au_20_. We calculate Φ_M_ of the Au_19_ and Au_20_NPs as 4.81 and 5.21 eV, respectively. These work functions are both lower than that of the pristine TiO_2_ surface, preserving downward band bending at the interface. However, both AuNP work functions are higher than Φ_S_ of the V_O_ defect TiO_2_ surface, yielding a positive ΔΦ, which in turn results in upward band bending and the formation of a Schottky barrier.

### 3.3 DFT Optimized AuNP–TiO_2_ Heterojunctions

To investigate the electronic properties of AuNP–TiO_2_ interfaces and their dependence on the AuNP and TiO_2_ surface defect characteristics, we construct four different types of heterojunctions. They consist of the four material combinations created by attaching open-shell Au_19_NPs (created via Wulff construction) or closed-shell, “magic” Au_20_NPs to either pristine or V_O_ defect TiO_2_ surfaces. Structure representation of the AuNPs are shown in Figure S2.1, while the AuNP–TiO_2_ heterojunctions are shown in Figure S5.1. Our 0 K DFT calculations illuminate how nanoparticle shape and open/closed shell nature impact interfacial electronics properties. They also highlight how oxygen vacancies on TiO_2_ surfaces affect NP electronic structure, adsorption behavior, and interfacial charge redistribution.

In the following, we use a DOS analysis to reveal how interactions at the AuNP–TiO_2_ interface determine the electronic structure by examining the wave function character in terms of AuNP and TiO_2_ dominated states. In the Au_19_NP–Pristine TiO_2_ heterojunction (Figure 3a), Au states dominate the HOMO while TiO_2_ states primarily form the LUMO, resulting in a HOMO–LUMO gap of 0.12 eV. Orbital-resolved charge density plots confirm that the HOMO is mainly localized on the Au atoms (98% Au character based on wave function analysis), with minor delocalization into the TiO_2_ surface layer (2% TiO_2_ character). Conversely, the LUMO extends across the entire TiO_2_ slab (100% TiO_2_ character). Meanwhile, the Au_20_NP–Pristine TiO_2_ heterojunction (Figure 3c) displays a markedly different DOS despite a similarly sized AuNP. The closed-shell nature in Au_20_NP–Pristine TiO_2_ leads to a much larger HOMO–LUMO gap of 1.33 eV. Both the HOMO (93% Au character) and LUMO (58% Au character) are predominantly localized on the Au_20_NP, with minor charge leakage into the top TiO_2_ layer (layer 1) in the LUMO (Figure 3c). To further illustrate the Au and TiO_2_ contributions to the DOS, a color-coded stick plot of the orbital energies at the Γ-point, overlaid with the DOS plot of a limited region around the band edge states, is shown in Figure S8.1.

**Figure 3:**
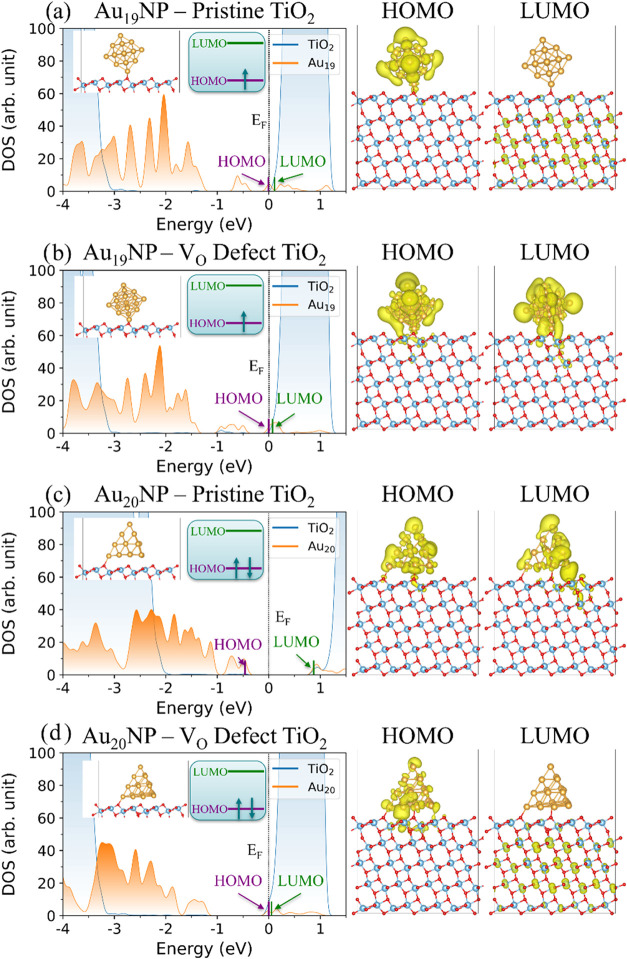
DOS of the AuNP–TiO_2_ heterojunction at the Γ-point and 0 K for: (a) Au_19_NP*–*Pristine TiO_2_, (b) Au_19_NP*–*V_O_ defect TiO_2_, (c) Au_20_NP*–*Pristine TiO_2_, and (d) Au_20_NP*–*V_O_ defect TiO_2_. *E*_F_ is set to zero for these DOS plots. Sections of the AuNP–TiO_2_ heterojunction structures containing the AuNPs and the first layer of TiO_2_ are shown as inserts, while the full structures can be found in Figure S5.1 The electron occupancies of the HOMO (purple) and LUMO (green) levels are also shown as inserts. Detailed contributions to decomposed eigen orbital energies can be found in Figure S8.1 whereas detailed Au DOS contributions to CB states are shown in Figure S11.1. Orbital-decomposed charge density plots at the HOMO and LUMO levels for each system at the Γ-point at 0 K are shown on the right. Note that these correspond to filled levels for the HOMO and unfilled levels for the LUMO orbitals.

Next, we examine how oxygen vacancies affect the electronic structure of the AuNP–TiO_2_ heterojunction. As shown in Figure S5.1, the presence of a V_O_ defect induces structural relaxation at the interface, altering the adsorption geometry of the AuNPs. For the Au_19_NP, the nanoparticle sits 1.2 Å closer to the defect TiO_2_ surface compared to the pristine case. In contrast, the Au_20_NP shows a much smaller shift of 0.1 Å upon the introduction of the same defect. This difference likely arises from the intrinsic geometry of the nanoparticles: the Au_20_ cluster has a broader contact area with the TiO_2_ surface, reducing its sensitivity to local defect-induced distortions.

To augment our analysis of the observed structural change induced by the V_O_, we calculate the adsorption energies (*E*_ads_) of the AuNPs, which are summarized in Table 1. *E*_ads_ is defined as the energy difference between the heterojunction and its isolated components

2 $$E_{ads}=E_{AuNP+TiO_{2}\;slab}^{heterojunction}-\left(E_{AuNP}^{isolated}+E_{TiO_{2}\;\mathrm{sl}ab}^{isolated}\right)$$

A more negative *E*_ads_ value denotes a stronger adsorption to the surface. For the pristine TiO_2_ surface, adsorption energy values of *E*_ads_ = −0.41 eV and *E*_ads_ = −0.92 eV are found for Au_19_NP and Au_20_NP, respectively. Au_20_NP shows a higher adsorption affinity to the pristine surface than Au_19_NP, due to a larger contact area with the TiO_2_ surface.

**Table 1:** Adsorption Energies *E*_ads_ of AuNPs within the 0 K DFT-Optimized AuNP–TiO_2_ Heterojunctions, and Static HOMO–LUMO Gaps of the Heterojunctions at the Γ-Point at 0 K

AuNP–TiO_2_ heterojunction	adsorption energy (eV)	HOMO–LUMO gap (eV)
Au_19_NP–Pristine TiO_2_	–0.41	0.12
Au_19_NP–V_O_ defect TiO_2_	–3.92	0.09
Au_20_NP–Pristine TiO_2_	–0.92	1.33
Au_20_NP–V_O_ defect TiO_2_	–2.18	0.06

We further examine how the V_O_ defect on the TiO_2_ surface enhances AuNP adsorption. For Au_19_NP, the magnitude of *E*_ads_ increases dramatically from *E*_ads_ = −0.41 eV to *E*_ads_ = −3.92 eV, an almost 10-fold increase on the V_O_ defect surface. Similarly, for the Au_20_NP–V_O_ defect TiO_2_ heterojunction, *E*_ads_ increases from *E*_ads_ = −0.92 eV to *E*_ads_ = −2.18 eV (a 2.4-fold increase). The increased magnitude of *E*_ads_ values on the V_O_ defect surface place both the Au_19_NP and the Au_20_NP V_O_ defect heterojunctions firmly within the chemisorption regime.^73,74^ These substantial values of *E*_ads_ demonstrate that oxygen vacancies serve as active anchoring sites, significantly strengthening AuNP–TiO_2_ interactions, consistent with previous findings for other Au clusters.^32,33^

While both the structure change and the change of *E*_ads_ are more dramatic in the Au_19_NP system upon the introduction of V_O_, the narrowing of the HOMO–LUMO gap is more pronounced in the Au_20_NP system. Specifically, the HOMO–LUMO gap decreases from 1.33 to 0.06 eV for Au_20_NP – V_O_ defect TiO_2_ (a 22-fold reduction), and from 0.12 to 0.09 eV for Au_19_NP – V_O_ defect TiO_2_ (a 25% reduction). DOS plots for these systems are shown in Figure 3b,d. This trend, while initially counterintuitive, underscores that changes in electronic structure are not solely dictated by structural proximity nor adsorption strength. Instead, the interaction between the nanoparticle’s electronic states and the V_O_ induced trap state plays a dominant role.

As previously established in Figure 1c,d for bare TiO_2_, the V_O_ introduces a shallow, filled trap state that raises the HOMO and shifts the Fermi level closer to the LUMO. A similar electronic signature change is observed for the Au_20_NP–TiO_2_ heterojunction upon comparison of Figure 3c,d, where the presence of the V_O_ defect markedly elevates the HOMO, consistent with the formation of a localized defect state at the interface.

In contrast to the dramatic impact of the oxygen defect on the DOS of the Au_20_NP-TiO_2_ heterojunctions, the overall DOS profile of the Au_19_NP–V_O_ defect TiO_2_ heterojunction (Figure 3b) closely resembles the DOS profile of the Au_19_NP–pristine TiO_2_ heterojunction (Figure 3a). This similarity originates from the intrinsic open-shell character of Au_19_NP itself, which lacks a clear bandgap (Figure S9.1a). The HOMO–LUMO gap is small (≤0.12 eV) for both the Au_19_NP–pristine and Au_19_NP–V_O_ defect heterojunctions, suggesting that the gap is dominated by the Au_19_NP rather than the surface defect (or the lack thereof). Upon first glance, however, a notable difference appears to emerge in the character of the LUMO. In the Au_19_NP–pristine TiO_2_ heterojunction, the LUMO is localized on TiO_2_ (0% Au wave function character) (Figure 3a), while in the Au_19_NP–V_O_ defect TiO_2_ system, the LUMO becomes localized on the AuNP with 80% Au wave function character (Figure 3b). Yet, upon closer inspection, we find that the LUMO and LUMO+1 states in the Au_19_NP–V_O_ defect TiO_2_ are separated by only 4 meV, i.e., they are essentially degenerate, and that the LUMO+1 state of the V_O_ defect system closely resembles the LUMO state of the pristine system. The LUMO+1 state is strongly localized on TiO_2_, with only 3% Au character, see Figure S10.1.

Next, we extend the Bader analysis of the TiO_2_ slabs discussed in Section 3.1 to the four AuNP–TiO_2_ interface models (Table S12.2). We observe clear correlations between the interfacial charge redistribution indicated by the Bader analysis of the slab models and the formation of a Schottky barrier in the bulk materials discussed in Section 3.2. The Au_20_NP–pristine TiO_2_ interface shows positive charge accumulation on the AuNP (+0.17 *e*) and negative charge accumulation on Layer 1 and 2 of the TiO_2_ surface (−0.02e and −0.10e, respectively). Electrons injected into a negatively charged TiO_2_ will readily return to the AuNP due to local electrostatic repulsion, leading to a short lifetime. This scenario is consistent with the downward band bending predicted for the pristine heterojunction in Section 3.2. In contrast, introducing the V_O_ defect to the Au_20_NP–TiO_2_ system inverts the charge distribution. The AuNP now accumulates a negative charge (−0.26 *e*), while the V_O_ defect TiO_2_ surface exhibits a net positive charge (+0.63e for Layer 1 and −0.20e for Layer 2, which adds up to +0.43e). Electrons injected into a positively charged TiO_2_ experience the local electrostatic attraction and are not expected to readily return to the AuNP. This reversal of the interfacial charge distribution and of the expected behavior of an electron injected from the AuNP into the TiO_2_ substrate, agrees with the finding in Section 3.2 that the introduction of V_O_ leads to the formation of a Schottky barrier and upward band bending in the Au_20_NP–V_O_ TiO_2_ system.

In the Au_19_NP–pristine TiO_2_ heterojunction, the charge redistribution is less pronounced. The Au_19_NP carries a slight negative charge (−0.02e), while the TiO_2_ surface is slightly positive overall (+0.19e for Layer 1 and −0.15e for Layer 2, which adds up to +0.04e). This subtle charge separation leads to an ambiguous scenario where we cannot decisively determine whether a Schottky barrier is formed from Bader charge analysis alone. In contrast, the Au_19_NP–V_O_ TiO_2_ heterojunction exhibits significant charge separation, with the AuNP acquiring a large negative charge (−0.64e), and a large positive charge on the TiO_2_ surface (+0.85e for Layer 1 and −0.16e for Layer 2, which adds up to +0.69e). This direction and magnitude of charge separation supports the formation of a Schottky barrier in the Au_19_NP–V_O_ TiO_2_ heterojunction, consistent with the upward band bending picture discussed in Section 3.2. Additional Bader Charge analysis result for the AIMD snapshots will be further discussed in the following Section 3.4.

### 3.4 Ab Initio Molecular Dynamics of AuNP–TiO_2_ Heterojunction

To investigate AuNP–TiO_2_ interfacial behavior under experimentally relevant conditions, we perform AIMD simulations at 300 K for all heterojunction systems. Upon heating, Au_19_NP loses its highly symmetrical shape on both the pristine surface (Figure S13.1a) and the V_O_ defect surface (Figure S13.1b). In contrast, Au_20_NP retains its shape more effectively, as illustrated in Figure S13.1c and S13.1d. Structural distortions of the AuNPs are further highlighted by comparing their configurations at 0K and 300 K, as shown in Figure S13.2. To better quantify these trends, we calculate the average root-mean-square deviation (RMSD) of nuclear positions for each heterojunction, using the average nuclear positions as the reference. The results are presented in Table 2. The RMSD including all Au and TiO_2_ atoms for the 4 systems studied are 0.30 Å for Au_19_NP on pristine and V_O_ defect TiO_2_ surfaces, 0.18 Å for Au_20_NP–pristine TiO_2_, and 0.16 Å for Au_20_NP–V_O_ defect TiO_2_, respectively. We observe that Au_19_NP heterojunctions exhibit greater motion than the Au_20_NP heterojunctions. Decoupling the average motion into different atomic groups reveals that the majority of the motion originates from the Au atoms. Specifically, the RMSD of Au atoms in Au_19_NP is an order of magnitude larger than that of the TiO_2_ slab. In contrast, for Au_20_NP, the Au atom motion is approximately four times greater than that of the TiO_2_ slab. The slightly reduced motion in the Au_20_NP–V_O_ defect heterojunction compared to its pristine counterpart, is attributed to a single Au atom filling the vacancy site and anchoring the Au_20_NP throughout the trajectory (Figure S13.1d). Although a Au atom fills the vacancy site in both AuNP defect systems (Figure S13.1c,d), the remaining Au atoms in Au_19_NP are highly flexible during the AIMD trajectory, leading to a higher RMSD for the Au atoms (1.37 Å) compared to Au_20_NP (0.53 Å).

**Table 2:** Average RMSD (Å) of the Atomic Positions for the AuNP–TiO_2_ Heterojunctions along the 6 ps, 300 K NVE AIMD Trajectory

average RMSD (Å)	Au_19_NP pristine TiO_2_	Au_19_NP V_O_ defect TiO_2_	Au_20_NP pristine TiO_2_	Au_20_NP V_O_ defect TiO_2_
All	0.30	0.30	0.18	0.16
Au	1.36	1.37	0.57	0.53
TiO_2_	0.13	0.13	0.14	0.12
Layer-1 TiO_2_	0.16	0.16	0.19	0.15
Layer-2 TiO_2_	0.12	0.12	0.13	0.12
Layer-3 TiO_2_	0.00	0.00	0.00	0.00
Layer-4 TiO_2_	0.13	0.13	0.13	0.12
Layer-5 TiO_2_	0.17	0.16	0.16	0.15

A key question is how the nuclear motion at elevated temperatures affects the electronic properties of the heterojunctions, which ultimately define their photocatalytic properties. We therefore examine the electronic structure changes along the AIMD trajectories by analyzing the DOS at arbitrarily selected times: 1 fs, 1.5 ps, 3 ps, 4.5 ps, and 6 ps (Figure 4). The DOS snapshots exhibit significant dynamic fluctuations, indicating pronounced sensitivity of the electronic states to structural rearrangements at room temperature. Comparing the dynamically evolving DOS from the AIMD trajectories at 300 K to the static, DFT-optimized DOS at 0K, several trends emerge.

**Figure 4:**
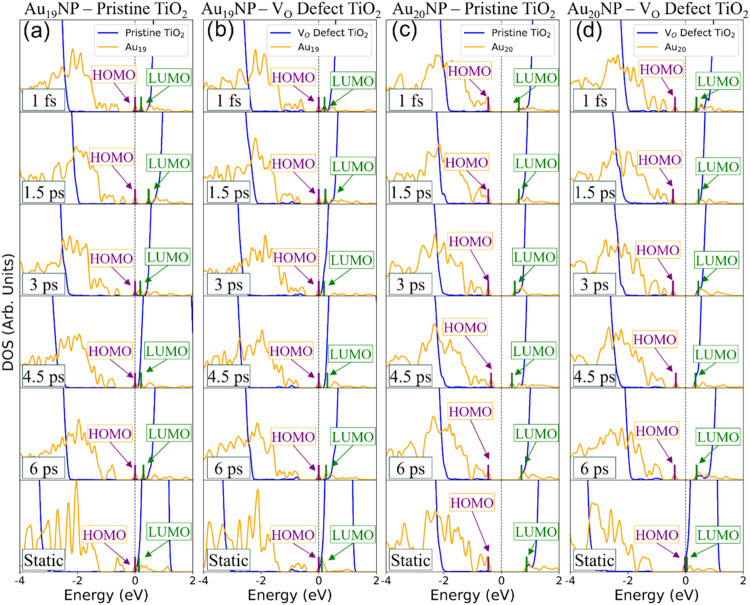
Calculated DOS for times 1 fs, 1.5 ps, 3 ps, 4.5 ps, 6 ps (from top to bottom) along the 6 ps, 300 K NVE AIMD trajectory, compared to the 0 K DFT static geometry at the Γ-point (bottom). Columns correspond to (a) Au_19_NP*–*Pristine TiO_2_, (b) Au_19_NP*–*V_O_ defect TiO_2_, (c) Au_20_NP*–*Pristine TiO_2_, and (d) Au_20_NP*–*V_O_ defect TiO_2_. *E*_F_ is set to zero for all plots. The HOMO and LUMO levels are shown as purple and green lines, respectively.

First, the HOMO–LUMO gap increases in all systems for the higher temperature except for the Au_20_NP–pristine TiO_2_. Table S13.1 lists the gap values for each time, along with the average over the entire 6 ps trajectory and the corresponding standard deviation. In Au_19_NP–pristine TiO_2_, the HOMO–LUMO gap increases 2.4-fold, from 0.12 eV in the DFT-optimized static geometry at 0 K (Γ-point) to an average of 0.29 eV along the AIMD trajectory at 300 K. A similar increase is observed for Au_19_NP–V_O_ defect TiO_2_, where the gap increases 3-fold, from 0.09 to 0.27 eV. In contrast, Au_20_NP–pristine TiO_2_ exhibits a slight 20% decrease of the gap, from 1.33 to 1.04 eV, while Au_20_NP–V_O_ defect TiO_2_ exhibits a substantial 15-fold increase, from 0.06 to 0.92 eV. We observe that systems with lower initial DFT-optimized HOMO–LUMO gaps, particularly Au_20_NP–V_O_ defect TiO_2_, exhibit the greatest gap increases during AIMD. We assign the especially surprising behavior of the Au_20_NP–V_O_ defect TiO_2_ system to enhanced surface reorganization at finite temperatures. Specifically, thermal fluctuations allow one of the Au atoms to more effectively occupy the V_O_ site than in the 0 K DFT-optimized geometry. This Au atom remains anchored at the vacancy throughout the AIMD trajectory, effectively passivating the defect. The resulting ″vacancy filling″ stabilizes the HOMO states while it destabilizes the LUMO states (Figure 4d) when compared with the 0 K results (Figure 3d), thereby widening the HOMO–LUMO gap at 300 K compared to 0 K. A similar analysis for the HOMO–1–LUMO gap (see Table S13.2) shows that it remains largely unaffected by the temperature rise for both Au_20_NP–TiO_2_ systems, exhibiting only a ≤16% decrease, but it widens more notably by ∼60 to 70% for the open-shell Au_19_NP–TiO_2_ systems compared to the static 0 K (Γ-point) gap values.

In addition to examining the impact of a finite temperature on the nuclear motion and electronic DOS distributions of the heterojunctions, it is instructive to study the temporal evolution of the HOMO/LUMO characters in terms of their relative contributions from the gold and TiO_2_ components of the junctions. Figure S13.3 shows the relative contributions of the AuNP to the HOMO and LUMO wave functions at different points in time along the AIMD trajectory at 300 K, along with the contributions to the static states at 0 K. While the HOMO states are predominantly derived from Au with relatively stable characters, most of the LUMO states’ wave function characters vary much more substantially between Au and TiO_2_ dominated configurations. These changes are visualized in greater detail in Figure S13.4-S13.7, which show band-decomposed charge density plots of the HOMO and LUMO levels for the AIMD snapshots of the various heterojunction configurations. The LUMOs of Au_19_NP–pristine TiO_2_ (Figure S13.4) and Au_20_NP–V_O_ defect TiO_2_ (Figure S13.7) show increased Au character (>30%) in AIMD snapshots, compared to 0% Au LUMO character in the DFT-optimized geometries (Figure 3a,d).

Interestingly, the LUMO fluctuations are most pronounced during the AIMD trajectory of the Au_19_NP–V_O_ defect TiO_2_ system (Figure S13.4). At 0 K, the LUMO and LUMO+1 states are nearly degenerate (Figure S10.1), which enhances the dynamic nature of the electronic structure. However, this near-degeneracy is not consistently observed along the 300 K trajectory. Analysis of the LUMO and LUMO+1 energetics for selected AIMD snapshots (Figure S13.5a) reveals that these states are only near-degenerate in 2 out of the 5 cases examined (specifically at 3 and 4.5 ps). Furthermore, the Au_19_NP–V_O_ defect TiO_2_ system shows that the LUMO+1 level from the static calculation, which is localized on TiO_2_ states, becomes the LUMO state at 3 and 4.5 ps of the AIMD trajectory (Figure S13.5b). In contrast, the LUMO state at the 1 fs, 1.5 and 6 ps snapshots represent that the original LUMO state localized on the Au in the static calculation. In all charge density plots in Figure S13.4-S13.7 (except for the near-degenerate states in the Au_19_NP–V_O_ defect TiO_2_ system) TiO_2_ contributions are primarily localized at the surface, with minimal involvement from the bulk TiO_2_. This surface localization contrasts with the 0 K case (Figure 3), where LUMO charge distributions can be delocalized throughout all TiO_2_ layers.

To enhance our interpretation of interfacial charge redistribution and Schottky barrier behavior (Section 3.3), we performed more Bader charge analysis on AIMD snapshots at 300 K. Snapshots were extracted from the AIMD trajectories for times 1, 1500, 3000, 4500, and 6000 fs. The Bader charges were computed for each snapshot, and the averaged results are summarized in Table S14.1. A full account of all layer-resolved Bader charges and individual snapshot results is provided in Tables S14.2-S14.5.

The AIMD results show trends consistent with those obtained from the static 0 K DFT Bader charge calculations in Section 3.3 and from the comparison of the material work functions in Section 3.2. For both heterojunction systems with V_O_, negative charge accumulation on the AuNP and a net positive accumulation on the TiO_2_ surface layers (sum of the charge of Layers 1 and 2) is observed, consistent with the formation of a Schottky barrier. Conversely, the pristine systems exhibit positive charge accumulation on the AuNPs and a net negative charge on the TiO_2_ surface layers (sum charge of Layers 1 and 2) at finite temperature, indicative of downward band bending as described in Sections 3.2 and 3.3. These AIMD-based Bader Charge observations provide compelling evidence that oxygen vacancies are not merely incidental but are essential for the formation of Schottky barriers at the AuNP–TiO_2_ heterojunction. Without an V_O_, the interface instead exhibits downward band bending, highlighting the decisive role of oxygen vacancies in enabling effective charge separation and rectifying band bending behavior.

The AIMD simulations highlight the added insight gained by incorporating finite-temperature dynamics into the study of AuNP–TiO_2_ heterojunctions. At 300 K, we observe notable atomic and electronic structure fluctuations, including partly substantial variations in the HOMO–LUMO gap, electronic state localization (AuNP vs TiO_2_) and interfacial charge redistribution. These dynamic effects, such as vacancy passivation and evolving LUMO character, reveal the importance of thermal motion in modulating interfacial behavior. Interestingly, the qualitative band bending and Schottky barrier characteristics are similar for the static 0 K and dynamic 300 K calculations, despite stark differences in other characteristics. By complementing static DFT calculations with AIMD, a more complete picture emerges of the heterojunctions under operating conditions relevant for photocatalysis and other light-driven processes. The findings support the need for advanced modeling strategies for capturing the complex, temperature-dependent nature of real interfaces.

## 4 Conclusions

The DFT and AIMD simulations reveal that oxygen vacancies, nanoparticle size and shape, and structural dynamics shape the electronic properties of AuNP–TiO_2_ heterojunctions. The presence of a V_O_ defect on the anatase TiO_2_ (101) slab creates a localized trap state, significantly narrowing the HOMO–LUMO gap from 3.34 to 0.08 eV. This shallow trap state leads to an n-type character of the TiO_2_ slab, as observed in experiments. Conversely, the pristine TiO_2_ slab exhibits p-type character, suggesting that oxygen vacancies play an important role in accurately modeling the TiO_2_ behavior in practical applications.

Further analysis shows that V_O_ defects are important for establishing a Schottky barrier at the AuNP–TiO_2_ interface. A Bader charge analysis of the heterojunction models reveals the underlying charge distribution of the interface, showing a net negative charge accumulation on the AuNP that is characteristic of upward band bending in the V_O_ defect TiO_2_ substrate. This trend is further supported by the work function calculations on the isolated components, which also predict upward band bending toward the AuNP-TiO_2_ interface. In contrast, pristine heterojunction systems exhibit the opposite charge polarity, leading to downward band bending and preventing the formation of a Schottky barrier.

Finite-temperature AIMD simulations reveal substantial dynamic electronic structure fluctuations and atomic structure rearrangements under room temperature (300 K). Compared to the static 0 K DFT results, the HOMO–LUMO gap for all heterojunctions widens during the AIMD trajectories. This effect is most pronounced in the Au_20_NP–V_O_ defect TiO_2_ system, where the HOMO–LUMO gap widens, from 0.06 eV in the static geometry to an average of 0.92 eV at 300 K. We attribute this dramatic gap opening to the passivation of the oxygen vacancy, where a single Au atom moves to fill the vacancy site.

Collectively, these findings highlight that explicitly accounting for nanoparticle size and shape, surface defects, and thermal motion is important for understanding interfacial electronic structure properties of AuNP-TiO_2_ heterojunctions.

## Supplementary Material



## Notes

Details on the construction of the TiO_2_ slab models (Section S1), Au models (Section S2), and Au-TiO_2_ heterojunctions (Section S3); equations for DOS calculations (Section S4); DFT optimized AuNP–TiO_2_ heterojunctions structures (Section S5); charge density plots for isolated pristine and V_O_ defect TiO_2_ slab (Section S6); planar-averaged electrostatic potential for isolated pristine and Vo defect TiO_2_ slab (Section S7); contribution decomposed eigen band energies for AuNPs–TiO_2_ heterojunctions (Table S8); DOS plots for Au_19_NP and Au_20_NP (Section S9); near-degeneracy states for Au_19_NP–Vo defect TiO_2_ (Figure S10); Au DOS contributions for AuNP–TiO_2_ heterojunctions (Figure S11); Bader charge analysis of the DFT optimized structures (Section S12); details and results from the AIMD of the AuNP–TiO_2_ heterojunction (Section S13); Bader charge analysis of the AIMD structures (Section S14) (PDF)

## References

[1] Song R., Chi H., Ma Q., Li D., Wang X., Gao W., Wang H., Wang X., Li Z., Li C. (2021). Highly Efficient
Degradation of Persistent Pollutants with 3D Nanocone TiO2-Based Photoelectrocatalysis. J. Am. Chem. Soc..

[2] Fujishima A., Honda K. (1972). Electrochemical Photolysis
of Water at a Semiconductor Electrode. Nature.

[3] Schneider J., Matsuoka M., Takeuchi M., Zhang J., Horiuchi Y., Anpo M., Bahnemann D. W. (2014). Understanding
TiO2 Photocatalysis:
Mechanisms and Materials. Chem. Rev..

[4] Chen X., Mao S. S. (2007). Titanium Dioxide
Nanomaterials: Synthesis, Properties,
Modifications, and Applications. Chem. Rev..

[5] Makuła P., Pacia M., Macyk W. (2018). How To Correctly Determine the Band
Gap Energy of Modified Semiconductor Photocatalysts Based on UV–Vis
Spectra. J. Phys. Chem. Lett..

[6] Thakur N., Thakur N., Kumar A., Thakur V. K., Kalia S., Arya V., Kumar A., Kumar S., Kyzas G. Z. (2024). A Critical
Review on the Recent Trends of Photocatalytic, Antibacterial, Antioxidant
and Nanohybrid Applications of Anatase and Rutile TiO2 Nanoparticles. Sci. Total Environ..

[7] Bourikas K., Kordulis C., Lycourghiotis A. (2014). Titanium Dioxide
(Anatase and Rutile):
Surface Chemistry, Liquid–Solid Interface Chemistry, and Scientific
Synthesis of Supported Catalysts. Chem. Rev..

[8] Tan T. H., Scott J., Ng Y. H., Taylor R. A., Aguey-Zinsou K.-F., Amal R. (2016). Understanding Plasmon
and Band Gap Photoexcitation Effects on the
Thermal-Catalytic Oxidation of Ethanol by TiO2-Supported Gold. ACS Catal..

[9] Tada H., Mitsui T., Kiyonaga T., Akita T., Tanaka K. (2006). All-Solid-State
Z-Scheme in CdS–Au–TiO2 Three-Component Nanojunction
System. Nat. Mater..

[10] Wu F., Du Y., Lv S., Zhao C., Yang X. (2022). DFT Modeling of CO2
Adsorption and HCOO• Group Conversion in Anatase Au-TiO2-Based
Photocatalysis. ACS Omega.

[11] Zou X., Vadell R. B., Liu Y., Mendalz A., Drillet M., Sá J. (2022). Photophysical
Study of Electron and Hole Trapping in
TiO2 and TiO2/Au Nanoparticles through a Selective Electron Injection. J. Phys. Chem. C.

[12] Long R., Prezhdo O. V. (2014). Instantaneous Generation
of Charge-Separated State
on TiO2 Surface Sensitized with Plasmonic Nanoparticles. J. Am. Chem. Soc..

[13] Wang J., Kim S.-D., Lee J.-Y., Kim J.-S., Jang N., Kim H., Kim D.-Y., Nam Y., Han M., Kong S.-H. (2024). Photocatalytic
Oxidization Based on TiO2/Au Nanocomposite Film for the Pretreatment
of Total Phosphorus (TP). Appl. Sci..

[14] Berdakin M., Soldano G., Bonafé F. P., Liubov V., Aradi B., Frauenheim T., Sánchez C. G. (2022). Dynamical Evolution of the Schottky
Barrier as a Determinant Contribution to Electron–Hole Pair
Stabilization and Photocatalysis of Plasmon-Induced Hot Carriers. Nanoscale.

[15] Borgwardt M., Mahl J., Roth F., Wenthaus L., Brauße F., Blum M., Schwarzburg K., Liu G., Toma F. M., Gessner O. (2020). Photoinduced Charge Carrier Dynamics
and Electron Injection
Efficiencies in Au Nanoparticle-Sensitized TiO2 Determined with Picosecond
Time-Resolved X-Ray Photoelectron Spectroscopy. J. Phys. Chem. Lett..

[16] Ke W., Qin X., Vazquez Y., Lee I., Zaera F. (2024). Direct Characterization
of Interface Sites in Au/TiO2 Catalysts Prepared Using Atomic Layer
Deposition. Chem. Catal..

[17] Dang D. T.-X., Vu N. H., Vu T. T.-H., Thoai N., Kawazoe Y., Phan B. T., Nguyen-Manh D. (2023). Combined Density
Functional Theory
and Boundary Element Methods Study on Optical and Electronic Properties
of Interfacial Au/TiO2 Defects. Opt. Mater.
X.

[18] Hansson G. V., Flodström S. A. (1978). Photoemission
Study of the Bulk and Surface Electronic
Structure of Single Crystals of Gold. Phys.
Rev. B.

[19] De
Renzi V., Rousseau R., Marchetto D., Biagi R., Scandolo S., del Pennino U. (2005). Metal Work-Function
Changes Induced by Organic Adsorbates: A Combined Experimental and
Theoretical Study. Phys. Rev. Lett..

[20] Huber E. E. (1966). The effect of
mercury contamination on the work function
of gold. Appl. Phys. Lett..

[21] Kim J. W., Kim A., Hwang H. U., Kim J. H., Choi S., Koch N., Shin D., Zhao Z., Liu F., Choi M., Lee K. M., Park Y. (2023). Work Function Measurement by Ultraviolet
Photoelectron Spectroscopy: Versailles Project on Advanced Materials
and Standards Interlaboratory Study. J. Vac.
Sci. Technol. A.

[22] Zhao Z., Li Z., Zou Z. (2010). Surface Properties and Electronic Structure of Low-Index
Stoichiometric Anatase TiO2 Surfaces. J. Phys.:
Condens. Matter.

[23] German E., Faccio R., Mombrú A. W. (2018). Comparison
of Standard DFT and Hubbard-DFT
Methods in Structural and Electronic Properties of TiO2 Polymorphs
and H-Titanate Ultrathin Sheets for DSSC Application. Appl. Surf. Sci..

[24] Mansfeldova V., Zlamalova M., Tarabkova H., Janda P., Vorokhta M., Piliai L., Kavan L. (2021). Work Function
of TiO2 (Anatase, Rutile,
and Brookite) Single Crystals: Effects of the Environment. J. Phys. Chem. C.

[25] Kashiwaya S., Morasch J., Streibel V., Toupance T., Jaegermann W., Klein A. (2018). The Work Function of
TiO2. Surfaces.

[26] Li H., Guo Y., Robertson J. (2015). Calculation
of TiO2 Surface and Subsurface Oxygen Vacancy
by the Screened Exchange Functional. J. Phys.
Chem. C.

[27] Puthiyaparambath M. F., Samuel J. E., Chatanathodi R. (2024). Tailoring
Surface Morphology on Anatase
TiO2 Supported Au Nanoclusters: Implications for O2 Activation. Nanoscale Adv..

[28] Guo Z., Prezhdo O. V., Hou T., Chen X., Lee S.-T., Li Y. (2014). Fast Energy Relaxation
by Trap States Decreases Electron Mobility
in TiO2 Nanotubes: Time-Domain Ab Initio Analysis. J. Phys. Chem. Lett..

[29] Nam Y., Li L., Lee J. Y., Prezhdo O. V. (2019). Strong Influence of Oxygen Vacancy
Location on Charge Carrier Losses in Reduced TiO2 Nanoparticles. J. Phys. Chem. Lett..

[30] Wang S., Gao Y., Miao S., Liu T., Mu L., Li R., Fan F., Li C. (2017). Positioning
the Water Oxidation Reaction Sites in Plasmonic
Photocatalysts. J. Am. Chem. Soc..

[31] Koga H., Tada K., Okumura M. (2015). Density Functional Theory Study of
Active Oxygen at the Perimeter of Au/TiO2 Catalysts. J. Phys. Chem. C.

[32] Wan W., Nie X., Janik M. J., Song C., Guo X. (2018). Adsorption, Dissociation,
and Spillover of Hydrogen over Au/TiO2 Catalysts: The Effects of Cluster
Size and Metal–Support Interaction from DFT. J. Phys. Chem. C.

[33] Siemer N., Lüken A., Zalibera M., Frenzel J., Muñoz-Santiburcio D., Savitsky A., Lubitz W., Muhler M., Marx D., Strunk J. (2018). Atomic-Scale Explanation of O2 Activation at the Au–TiO2
Interface. J. Am. Chem. Soc..

[34] Farnesi
Camellone M., Zhao J., Jin L., Wang Y., Muhler M., Marx D. (2013). Molecular Understanding of Reactivity
and Selectivity for Methanol Oxidation at the Au/TiO2 Interface. Angew. Chem., Int. Ed..

[35] Farnesi
Camellone M., Marx D. (2013). On the Impact of Solvation on a Au/TiO2
Nanocatalyst in Contact with Water. J. Phys.
Chem. Lett..

[36] Huang J., He S., Goodsell J. L., Mulcahy J. R., Guo W., Angerhofer A., Wei W. D. (2020). Manipulating Atomic Structures at the Au/TiO2 Interface
for O2 Activation. J. Am. Chem. Soc..

[37] Byrne C., Ganguly P., Barbara Maccioni M., Nolan M., Hermosilla D., Merayo N., Blanco Á., Hinder S., Pillai S. C. (2024). Impact
of Au on the Transition Temperature and Photocatalytic Activity of
TiO2. J. Photochem. Photobiol. Chem..

[38] Wang, Y. ; Zhou, G. DFT Investigations of Aun Nano-Clusters Supported on TiO2 Nanotubes: Structures and Electronic Properties. Molecules 2022, 27 (9). 2756 10.3390/molecules27092756. 35566107 PMC9100182

[39] Ozawa K., Emori M., Yamamoto S., Yukawa R., Yamamoto S., Hobara R., Fujikawa K., Sakama H., Matsuda I. (2014). Electron–Hole
Recombination Time at TiO2 Single-Crystal Surfaces: Influence of Surface
Band Bending. J. Phys. Chem. Lett..

[40] Kresse G., Furthmüller J. (1996). Efficient
Iterative Schemes for Ab Initio Total-Energy
Calculations Using a Plane-Wave Basis Set. Phys.
Rev. B.

[41] Kresse G., Furthmüller J. (1996). Efficiency of Ab-Initio Total Energy
Calculations for
Metals and Semiconductors Using a Plane-Wave Basis Set. Comput. Mater. Sci..

[42] Perdew J. P., Burke K., Ernzerhof M. (1996). Generalized
Gradient Approximation
Made Simple. Phys. Rev. Lett..

[43] Blöchl P. E. (1994). Projector
Augmented-Wave Method. Phys. Rev. B.

[44] Kresse G., Joubert D. (1999). From Ultrasoft Pseudopotentials to the Projector Augmented-Wave
Method. Phys. Rev. B.

[45] Huang W., Ji M., Dong C.-D., Gu X., Wang L.-M., Gong X. G., Wang L.-S. (2008). Relativistic Effects
and the Unique Low-Symmetry Structures
of Gold Nanoclusters. ACS Nano.

[46] Sun K., Kohyama M., Tanaka S., Takeda S. (2015). Understanding of the
Activity Difference between Nanogold and Bulk Gold by Relativistic
Effects. J. Energy Chem..

[47] Anisimov V. I., Zaanen J., Andersen O. K. (1991). Band Theory
and Mott Insulators:
Hubbard U Instead of Stoner I. Phys. Rev. B.

[48] Krukau A. V., Vydrov O. A., Izmaylov A. F., Scuseria G. E. (2006). Influence of the
Exchange Screening Parameter on the Performance of Screened Hybrid
Functionals. J. Chem. Phys..

[49] Bartkowiak A., Korolevych O., Chiarello G. L., Makowska-Janusik M., Zalas M. (2021). How Can the Introduction
of Zr4+ Ions into TiO2 Nanomaterial Impact
the DSSC Photoconversion Efficiency? A Comprehensive Theoretical and
Experimental Consideration. Materials.

[50] Park K., Raman M., Olatunbosun A.-J., Pohlmann J. (2024). Revisiting DFT+U Calculations
of TiO2 and the Effect of the Local-Projection Size. AIP Adv..

[51] Curnan M. T., Kitchin J. R. (2015). Investigating the Energetic Ordering of Stable and
Metastable TiO2 Polymorphs Using DFT+U and Hybrid Functionals. J. Phys. Chem. C.

[52] Duan Z., Henkelman G. (2015). CO Oxidation
at the Au/TiO2 Boundary: The Role of the
Au/Ti5c Site. ACS Catal..

[53] Zheng M., Jia C., Sharman E., Jiang J., Fan W., Zhao X. (2021). Maximizing
the Synergistic Effect of PdAu Catalysts on TiO2(1 0 1) for Robust
CO2 Reduction: A DFT Study. Appl. Surf. Sci..

[54] Hamamoto N., Tatsumi T., Takao M., Toyao T., Hinuma Y., Shimizu K., Kamachi T. (2021). Effect of
Oxygen Vacancies on Adsorption
of Small Molecules on Anatase and Rutile TiO2 Surfaces: A Frontier
Orbital Approach. J. Phys. Chem. C.

[55] Harb M., Jeantelot G., Basset J.-M. (2019). Insights into the Most Suitable TiO2
Surfaces for Photocatalytic O2 and H2 Evolution Reactions from DFT
Calculations. J. Phys. Chem. C.

[56] Mieritz D. G., Renaud A., Seo D.-K. (2016). Unusual Changes in Electronic Band-Edge
Energies of the Nanostructured Transparent n-Type Semiconductor Zr-Doped
Anatase TiO2 (Ti1–xZrxO2; *x* < 0.3). Inorg. Chem..

[57] Cushing Scott. K., Meng F., Zhang J., Ding B., Chen C. K., Chen C.-J., Liu R.-S., Bristow A. D., Bright J., Zheng P., Wu N. (2017). Effects of
Defects on Photocatalytic
Activity of Hydrogen-Treated Titanium Oxide Nanobelts. ACS Catal..

[58] Wang X., Liu L., Cao H., Gong S., Pan H., Liu X., Wang P., Zhang Y. (2025). Constructing Heterojunction
Photocatalyst
Systems with Spatial Distribution of Au Single Atoms for CO2 Reduction. ACS Appl. Mater. Interfaces.

[59] Guo Y., Chen S., Yu Y., Tian H., Zhao Y., Ren J.-C., Huang C., Bian H., Huang M., An L., Li Y., Zhang R. (2019). Hydrogen-Location-Sensitive Modulation
of the Redox Reactivity for Oxygen-Deficient TiO2. J. Am. Chem. Soc..

[60] Zafar Z., Yi S., Li J., Li C., Zhu Y., Zada A., Yao W., Liu Z., Yue X. (2022). Recent Development in Defects Engineered
Photocatalysts: An Overview of the Experimental and Theoretical Strategies. Energy Environ. Mater..

[61] Tung R. T. (2014). The Physics
and Chemistry of the Schottky Barrier Height. Appl. Phys. Rev..

[62] Schottky W. (1942). Vereinfachte
Und Erweiterte Theorie Der Randschicht-Gleichrichter. Z. Phys..

[63] Li H., He D., Zhou Q., Mao P., Cao J., Ding L., Wang J. (2017). Temperature-Dependent Schottky Barrier in High-Performance Organic
Solar Cells. Sci. Rep..

[64] DuChene J. S., Sweeny B. C., Johnston-Peck A. C., Su D., Stach E. A., Wei W. D. (2014). Prolonged Hot Electron Dynamics in
Plasmonic-Metal/Semiconductor
Heterostructures with Implications for Solar Photocatalysis. Angew. Chem., Int. Ed..

[65] Arshad M. S., Trafela Š., Rožman K. Ž., Kovač J., Djinović P., Pintar A. (2017). Determination of Schottky
Barrier
Height and Enhanced Photoelectron Generation in Novel Plasmonic Immobilized
Multisegmented (Au/TiO2) Nanorod Arrays (NRAs) Suitable for Solar
Energy Conversion Applications. J. Mater. Chem.
C.

[66] Zhang Z., Yates J. T. (2012). Band Bending in Semiconductors: Chemical
and Physical Consequences at Surfaces and Interfaces. Chem. Rev..

[67] Sun Z., Fang Y. (2021). Electrical Tuning Effect
for Schottky Barrier and Hot-Electron Harvest
in a Plasmonic Au/TiO2 Nanostructure. Sci. Rep..

[68] Wang A., Wu S., Dong J., Wang R., Wang J., Zhang J., Zhong S., Bai S. (2021). Interfacial Facet Engineering on
the Schottky Barrier between Plasmonic Au and TiO2 in Boosting the
Photocatalytic CO2 Reduction under Ultraviolet and Visible Light Irradiation. Chem. Eng. J..

[69] Kryachko E. S., Remacle F. (2007). The Magic Gold Cluster Au20. Int. J. Quantum Chem..

[70] Li J., Li X., Zhai H.-J., Wang L.-S. (2003). Au20: A Tetrahedral Cluster. Science.

[71] Tarrat N., Rapacioli M., Cuny J., Morillo J., Heully J.-L., Spiegelman F. (2017). Global Optimization of Neutral and Charged 20- and
55-Atom Silver and Gold Clusters at the DFTB Level. Comput. Theor. Chem..

[72] Barmparis G. D., Lodziana Z., Lopez N., Remediakis I. N. (2015). Nanoparticle
Shapes by Using Wulff Constructions and First-Principles Calculations. Beilstein J. Nanotechnol..

[73] Glenna D. M., Jana A., Xu Q., Wang Y., Meng Y., Yang Y., Neupane M., Wang L., Zhao H., Qian J., Snyder S. W. (2023). Carbon
Capture: Theoretical Guidelines
for Activated Carbon-Based CO2 Adsorption Material Evaluation. J. Phys. Chem. Lett..

[74] Patel H. A., Byun J., Yavuz C. T. (2017). Carbon
Dioxide Capture Adsorbents:
Chemistry and Methods. ChemSusChem.

