# Supplementary material for: Electronic Structure of the Au Nanoparticle-TiO2 Heterojunction: Influence of Nanoparticle Size, Shape, Oxygen Vacancies, and Temperature
Source: J Phys Chem C Nanomater Interfaces. 2025 Dec 6;129(50):22021–32. doi: 10.1021/acs.jpcc.5c05449 (PMC12720240; doi:10.1021/acs.jpcc.5c05449)
Supplement: Supplementary file 1 [file jp5c05449_si_001.pdf]

Supplemental Information for:

Electronic Structure of the Au Nanoparticle-TiO_2 Heterojunction: Influence of Nanoparticle Size, Shape, Oxygen Vacancies, and Temperature

Carlos Mora Perez[1], Drew M. Glenna[1,2], Ernest Hermosillo[1], Zachery Donnellan[1,3], Soumyadeep Ghosh[1], Oliver Gessner[1], Jin Qian[1*]

1. Chemical Sciences Division, Lawrence Berkeley National Laboratory, Berkeley, California 94720, United States
2. Department of Nuclear Engineering & Industrial Management, University of Idaho, Idaho Falls, Idaho 83402, United States
3. Department of Chemistry, University of California, Berkeley, California 94720

*Corresponding Author: Jin Qian, jqian2@lbl.gov

Table of Contents

S1: Construction of the TiO_2 Slab Models 2

S2: Construction of the Au Models 5

S3: Construction of the Au-TiO_2 Heterojunctions 6

S4. DOS Equations 6

S5: DFT Optimized AuNP–TiO_2 Heterojunctions 7

S6: Charge Density Plots for Pristine and V_o Defect TiO_2 Slab 8

S7: Planar-Averaged Electrostatic Potential for Pristine and V_o Defect TiO_2 Slab 9

S8: Contribution Decomposed Eigen Band Energies for AuNPs–TiO_2 Heterojunctions 10

S9: DOS Plots for Au_{19}NP and Au_{20}NP 10

S10: Near-Degeneracy States for Au_{19}NP–V_o Defect TiO_2 11

S11: Au DOS Contributions for AuNPs–TiO_2 Heterojunctions 11

S12: Bader Charge Analysis of the DFT Optimized Structures 12

S13: Molecular Dynamics of AuNP–TiO_2 Heterojunction 13

S14: Bader Charge Analysis of the AIMD Structures 20

S15: References 23

S1: Construction of the TiO_2 Slab Models

The lattice parameters of bulk anatase TiO_2 are first optimized using DFT-PBE, resulting in parameter values of a, b = 3.81 Å and c = 9.77 Å. The optimized bulk TiO_2 is then cleaved along the (101) direction with oxygen termination. A 4 × 4 slab is constructed with a surface area of 20.97 × 15.24 Å^2, which is large enough to minimize lateral interactions between adsorbed Au_{19} or Au_{20} nanoparticles. A vacuum region of 30 Å in the c-direction is introduced to avoid interactions between periodic slab images.

To ensure accuracy, we test the convergence of the work function, Φ, and the HOMO–LUMO gap as a function of slab thickness by comparing 3-, 5-, and 7-layer systems (**Figure S1.1**). HOMO and LUMO states are obtained at the Γ-point. In each configuration, only the top two layers are allowed to relax structurally, while the bottom layers remain fixed to maintain bulk-like properties. The work function proves to be independent of slab thickness for pristine TiO_2. However, the bandgap decreases by 0.2 eV from 3-layer to 5-layer slabs and by 0.01 eV from 5-layer to 7-layer slabs, indicating electronic property convergence at five layers. Thus, a 5-layer slab is selected as our model for subsequent analyses.

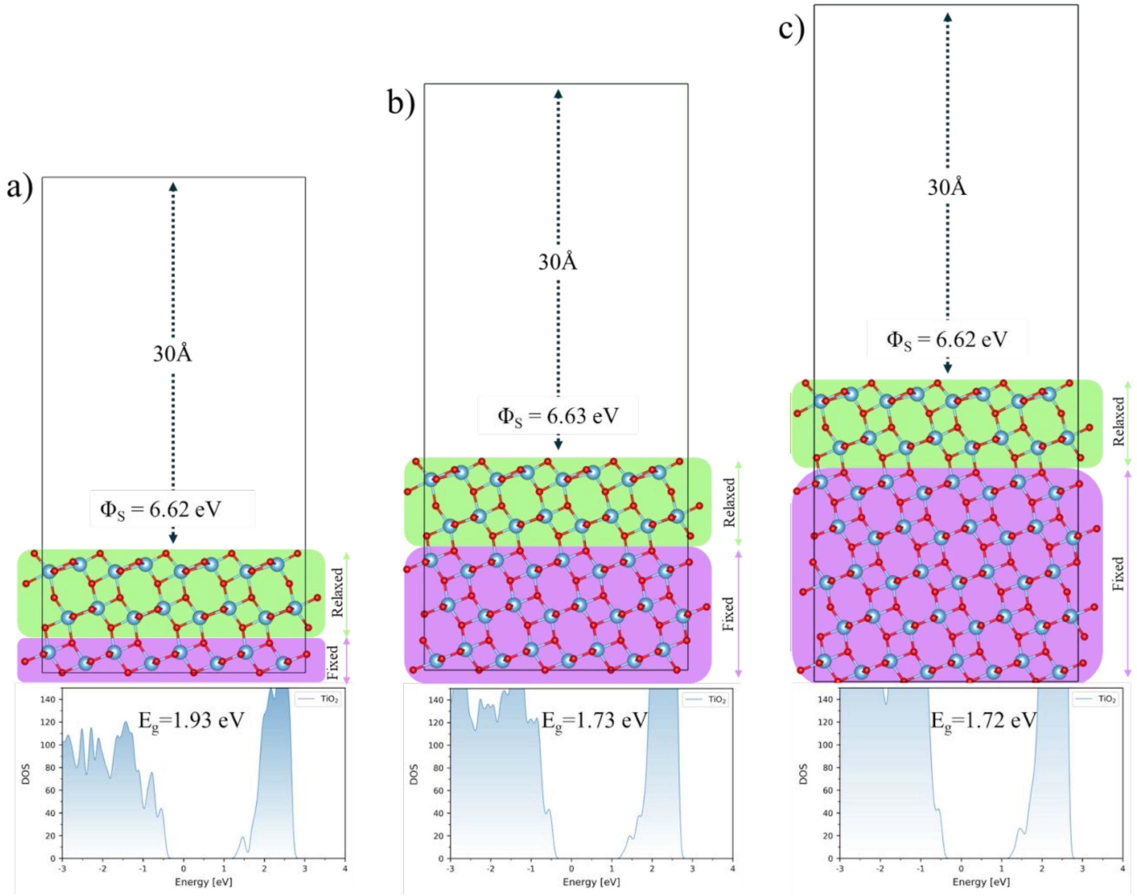

Figure S1.1. Pristine surface slab model of the anatase TiO_2 (101) surface with a 2x4 surface area (21.0 Å x 15.3 Å), a 30 Å vacuum region, and with various layer thicknesses at 0° K and 3x3x1 kpoint mesh. Only the top two layers were geometrically relaxed (highlighted in green), while the lower layers were kept fixed (highlighted in purple) to preserve bulk properties. a) 3-layer thickness. b) 5-layer thickness. c) 7-layer thickness. The work function of TiO_2 is not dependent on layer thickness, while the bandgap (+U correction not included) decreases with an increase in TiO_2 layers. The bandgap converged at 5-layer thickness.

When modeling surface adsorbates using DFT, two common slab modeling approaches are used: (1) constraining the slab by fixing some layers with the adsorbate placed on one side, and (2) placing adsorbates on both sides of a fully relaxed slab. The second method is computationally prohibitive for our system. Furthermore, the first method is widely used in TiO_2 surface studies.[1–6] Therefore, we proceeded with the first method. However, we note that the selection of fixed layers can significantly affect the electronic structure.

To assess this impact, we analyze the changes in work function, HOMO–LUMO gap, and total energy resulting from the position and depth of fixed bulk layers (**Figure S1.2**). When the surface layers (layers 1 and/or 5) are fixed (**Figures S1.2a** and **S1.2b**), the bandgap is artificially reduced to approximately 2.76 eV, due to the HOMO and LUMO states originating from constrained surface layers. In contrast, relaxing both surface layers (**Figures S1.2c** and **S1.2d**) results in a ~0.6 eV larger bandgap due to increased delocalization of the orbitals at the band edges. Additionally, the work function is highly sensitive to the selection of fixed layers, varying by up to ~0.7 eV.

Relaxing both surface layers (**Figures S1.2c** and **S1.2d**), rather than only one (**Figure S1.2b**), results in a more stable structural configuration with a total energy lowered by ~7 eV (162 kcal/mol). Allowing additional layers to relax further decreases the total energy. In **Figure S1.2d**, where only the central layer (layer 3) is fixed, it exhibits a 330 meV (7.59 kcal/mol) lower energy than **Figure S1.2c**. The configuration in **Figure S1.2d** is therefore preferred, as relaxing additional layers promotes better surface reorganization when accommodating an adsorbate, an essential requirement for accurately capturing the interfacial behavior in the Au–TiO_2 heterojunction. Therefore, the configuration in **Figure S1.2d**, is selected as the basis of our surface model.

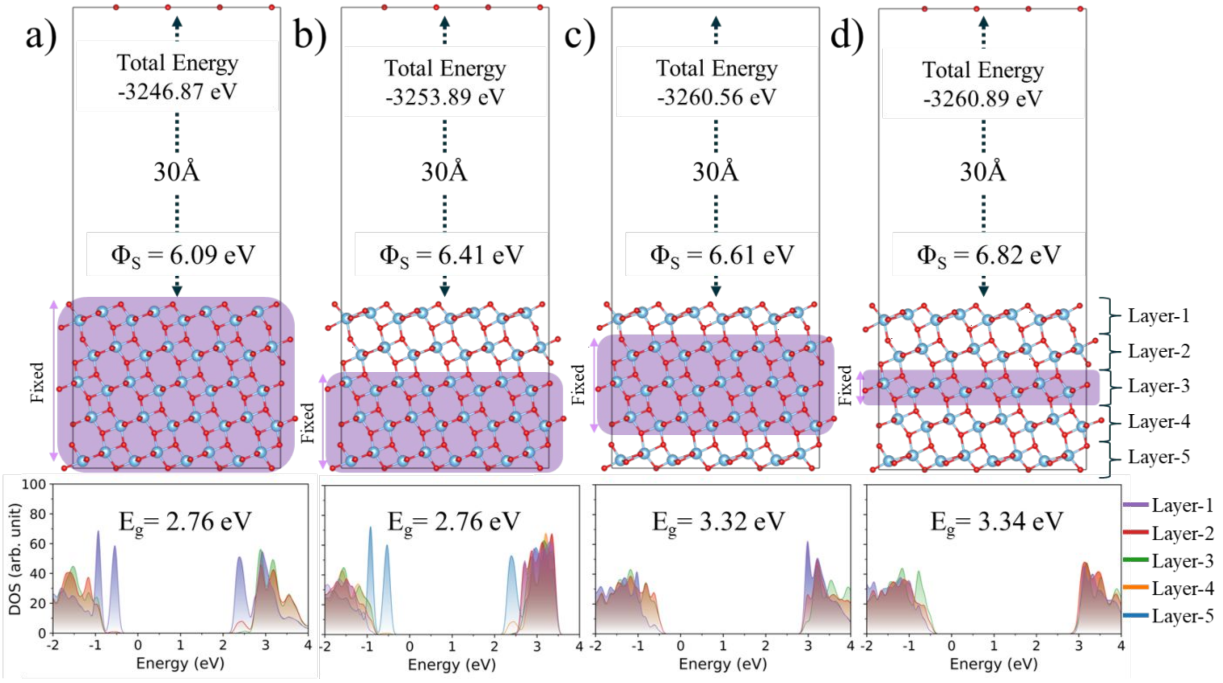

Figure S1.2. Pristine surface slab model of the anatase TiO_2 (101) surface, 5-layer thickness, 2x4 surface area (21.0 Å x 15.3 Å), and 30 Å vacuum region at 0 K. Layers 1 and 5 are exposed to the vacuum. Purple-shaded regions indicate the atomic position of fixed “bulk” layers, and all other layers are relaxed

geometrically. The layer-resolved DOS plots at the 3x3x1 kpoint mesh with +U corrections are shown below each slab model, with the bandgap values as inserts. A 6.9 eV +U correction term was added to Ti d electrons to replicate the experimental bandgap for all systems (see **Figure S1.3**). The total energy and work function (Φ) values are shown as inserts within the slab model cell. a) All layers are kept fixed (layers 1-5). b) The lowest three layers are fixed (layers 3-5). c) Three center layers are fixed (layers 2-4). d) The centermost layer was kept fixed (layer 3). We observed that if surface layers (1 and/or 5) are kept fixed, such as in a), where both surface layers are fixed, and b) one surface layer is fixed, the bandgap is artificially small (2.76 eV). Band resolved DOS plots further reveal that this artifact is because the HOMO and LUMO originate from frozen surface layers. In case (a), the HOMO & LUMO are localized on layers 1&5, while in (b), the HOMO & LUMO are localized on layer 5, as seen by the layer resolved DOS plots. However, when both surface layers (1 and 5) are relaxed, such as in c & d, the bandgap is ~0.6 eV larger. The work function is highly sensitive to the placement of the fixed "bulk" layers, fluctuating up to ~0.7 eV. Additionally, when both surface layers (1 and 5) are relaxed (c & d) rather than a single surface layer (b), the total energy of the system is stabilized by ~7 eV. Thus, both surface layers (1 and 5) must be relaxed to reach a more converged total energy. Furthermore, the more layers that are allowed to relax, the lower the total energy. Comparing c & d, a decrease of 330 meV in total energy is observed. Overall, the computational setup in d) allows for the best representation of electronic properties (Φ, bandgap, and total system energy). Thus, a single centermost layer (layer 3) is chosen as the basis for all calculations.

All energies reported in **Figure S1.2** were obtained using a simplified, rotationally invariant DFT+U approach. A U value of 6.9 eV was applied to Ti 3d orbitals, selected to reproduce the experimental bulk TiO_2 bandgap of 3.2 eV[7] (**Figure S1.3**). Without the +U correction, the bulk TiO2 bandgap is significantly underestimated by ~1 eV. While this +U correction was calibrated for the 3D bulk crystal, the reduced dimensionality of the surface slab results in a modified bandgap. Specifically, the **Figure S1.2d** 2D surface slab structure exhibits an increased gap of 0.14 eV (3.34 eV gap) compared to the 3D bulk.

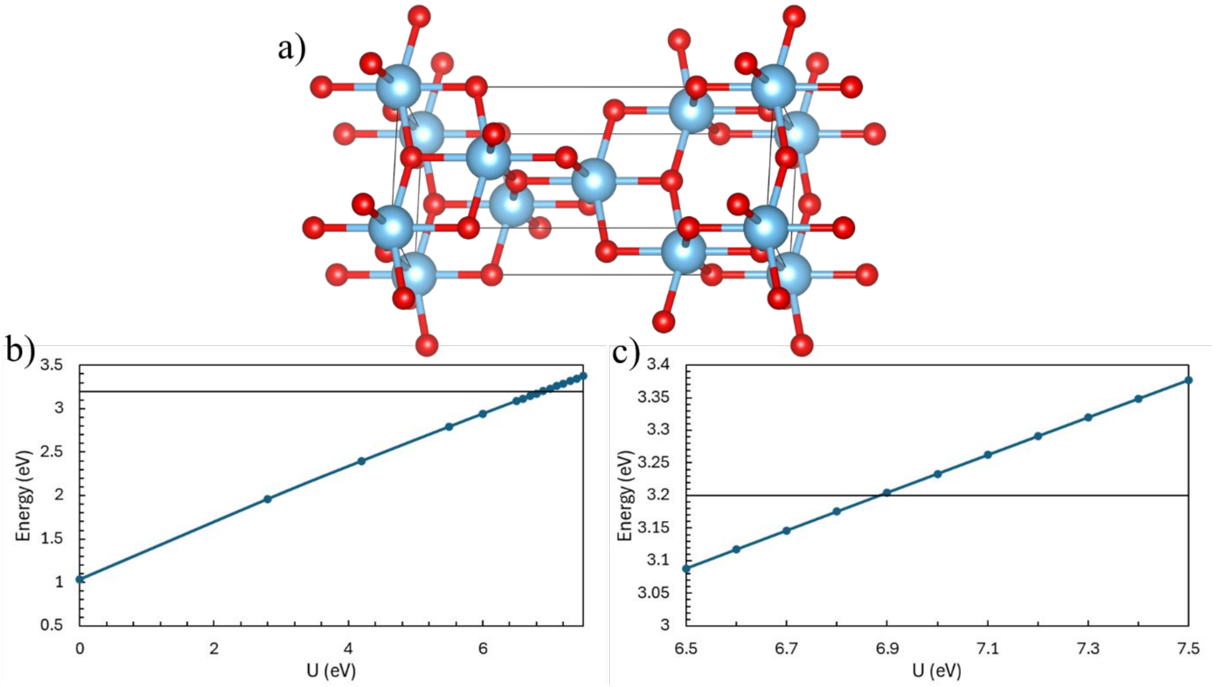

Figure S1.3. a) Structure model of the 3D bulk anatase TiO_2 crystal at 0 K. b) The bandgap value of bulk anatase TiO_2 crystal with varying Hubber U value added to the Ti 3d states to model a realistic bandgap. The horizontal line represents the experimental bandgap of 3.2 eV[7] at room temperature for bulk anatase TiO_2. c) Zoomed in on a region near the experimental bulk bandgap around 3.2 eV. A U value of 6.9 eV is chosen to replicate the experimental bulk bandgap best.

S2: Construction of the Au models

We investigate several distinct gold model structures. First, we build Wulff-constructed nanoparticles[8] based on bulk Au lattice parameters and a low-energy (111) surface facet to minimize total surface energy. Specifically, we construct an open-shell Wulff nanoparticle containing 19 Au atoms with a radius of approximately 6.6 Å, with octahedral (O_h) symmetry. Second, we consider the similarly sized Au_{20} "magic" cluster[9], known for its tetrahedral (T_d) symmetry, high stability, and closed-shell electronic structure. This cluster is the lowest-energy isomer for 20 Au atoms[10,11], possesses a large HOMO–LUMO gap (~1.8 eV[9,10]), and accurately reproduces bulk Au–Au bond distances.[9–11] Au_{19}NP and Au_{20}NP structures are shown in **Figure S2.1**. Finally, we construct an Au (111) surface slab analogous to the pristine TiO_2 (101) surface, consisting of five atomic layers with the central layer fixed to bulk positions. The Au (111) slab has a 2×2 surface area (11.8 Å × 10.2 Å) and a vacuum region of 30 Å to prevent interactions between periodic images.

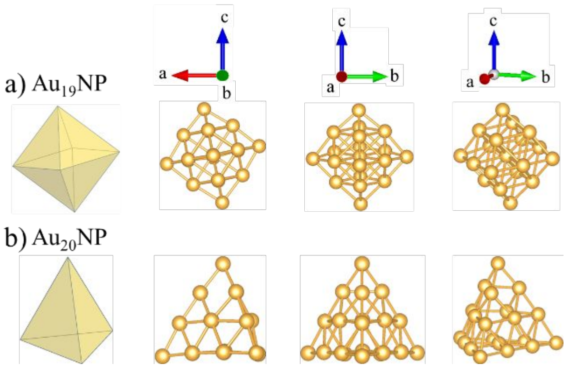

Figure S2.1. Structure representation of a) the Au_{19}NP Wulff-constructed nanoparticle with octahedral (O_h) symmetry and b) the Au_{20}NP "magic" cluster with tetrahedral (T_d) symmetry.

S3: Construction of the Au-TiO_2 heterojunctions

Building on our insights from the Au and TiO_2 systems, we then investigate the Au–TiO_2 interface. Our calculations show that a V_O defect is critical for reproducing the correct interfacial band bending (see Section 3b) and introduces a localized trap state (see Sections 3a & S7). Based on this understanding, in all defect-containing models the AuNP was positioned centered above the surface V_O defect, created by removing a bridging oxygen atom. Alternative adsorption sites were not explored, as the values of E_{ads} confirm a strong chemisorption bond at the V_O defect site, making this a dominant and energetically favorable binding site. For the pristine TiO_2 surface, the AuNPs were placed at the analogous bridging oxygen location to allow a direct comparison. The nanoparticles were oriented to maximize surface contact, and to preserve their overall shape upon optimization. This approach allowed us to distinguish the effect of the vacancy on the interfacial electronic structure from other potential structural factors.

Due to the high computational cost of heterojunction models, we limit our study to two AuNPs (Au_{19} and Au_{20}) on the pristine and V_O defect TiO_2 surfaces. In each case, the AuNP is initially placed 2 Å away from the topmost layer of the optimized TiO_2 slab. The entire heterojunction model is then reoptimized, with the exception of the central layer of the TiO_2 slab, which remains fixed at its bulk positions, as detailed in the previous **SI Section S1**. This approach yields four unique heterojunctions: (1) Au_{19} NP on the pristine TiO_2 (101) surface (**Figure S5.1a**), (2) Au_{19} NP on the V_O defect TiO_2 (101) surface (**Figure S5.1b**), (3) Au_{20} NP on the pristine TiO_2 (101) surface (**Figure S5.1c**), and (4) Au_{20} NP on the V_O defect TiO_2 (101) surface (**Figure S5.1d**).

S4. DOS Equations

The Total DOS is described as:

$$Total\ DOS(E) = \sum_i \delta(E - \varepsilon_i) \quad (1)$$

where ε_i is the eigenvalue of the eigenstate $|\psi_i\rangle$. The projected DOS (pDOS), which represents the contributions of specific atomic subsets to the total DOS, is described as:

$$pDOS_j(E) = \sum_i \left|\langle \phi_j \mid \psi_i \rangle\right|^2 \delta(E - \varepsilon_i) \quad (2)$$

where the eigenstate $|\psi_i\rangle$ is projected onto your chosen orthonormal basis set $|\phi_j\rangle$. For simplicity, we refer to all DOS and pDOS plots as DOS plots. In our DOS plots the function $(\delta(E-\varepsilon_i))$ is replaced by a gaussian function as:

$$\delta(E-\varepsilon_i) = \frac{1}{\sigma\sqrt{2\pi}} e^{\frac{(E-\varepsilon_i)^2}{2\sigma^2}} \tag{3}$$

where σ is the smearing parameter and determines the width of the Gaussian peak. In all cases σ is set to 0.05 eV. This smearing parameter results in the extension of the DOS curve beyond the calculated eigenvalues.

S5: DFT Optimized AuNP–TiO_2 Heterojunctions

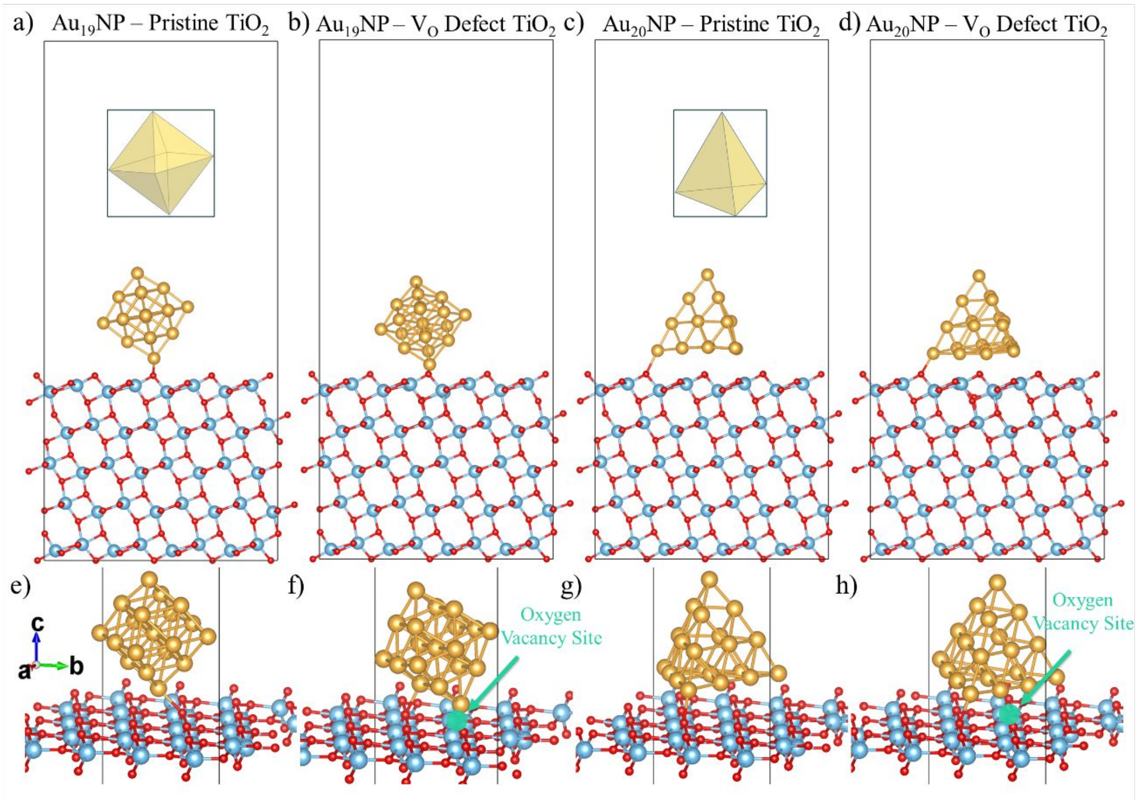

Figure S5.1. Optimized structure models for a) the Au_{19} Wulff constructed nanoparticle (NP) on the pristine anatase TiO_2 (101) surface, the centroid of Au_{19}NP to the surface layer of TiO_2 distance is 5.41 Å. b) the Au_{19}NP on the oxygen vacancy (V_O) TiO_2 (101) surface, the Au_{19}NP-TiO_2 surface distance is 4.17 Å. The centroid of the Au_{19}NP is 26% (1.2 Å) closer to the defect TiO_2 surface than on the pristine surface, c) the Au_{20} "magic" cluster on the pristine TiO_2 (101) surface, the Au_{20}NP-TiO_2 surface distance is 4.05 Å, and d) the Au_{20}NP on the V_O Defect TiO_2 (101) surface, the Au_{20}NP-TiO_2 surface distance is 3.95 Å. The centroid of the Au_{20}NP is 3% (0.1 Å) closer to the defect TiO_2 surface than on the pristine surface at 0 K. Cartoon representation of the Au_{19}NP and Au_{20}NP are shown as inserts in a) and c), respectively. Angled view of the optimized structures, showing only the uppermost TiO_2 layer (layer 1), highlight the differences of the AuNP location between pristine and defect surfaces for e) Au_{19}NP and

pristine-TiO_2, f) Au_{20}NP–pristine-TiO_2, g) Au_{19}NP–V_O defect TiO_2, and h) Au_{20}NP–V_O defect TiO_2 heterojunctions. Oxygen vacancies are shown in green.

S6: Charge Density Plots for Pristine and V_O Defect TiO_2 Slab

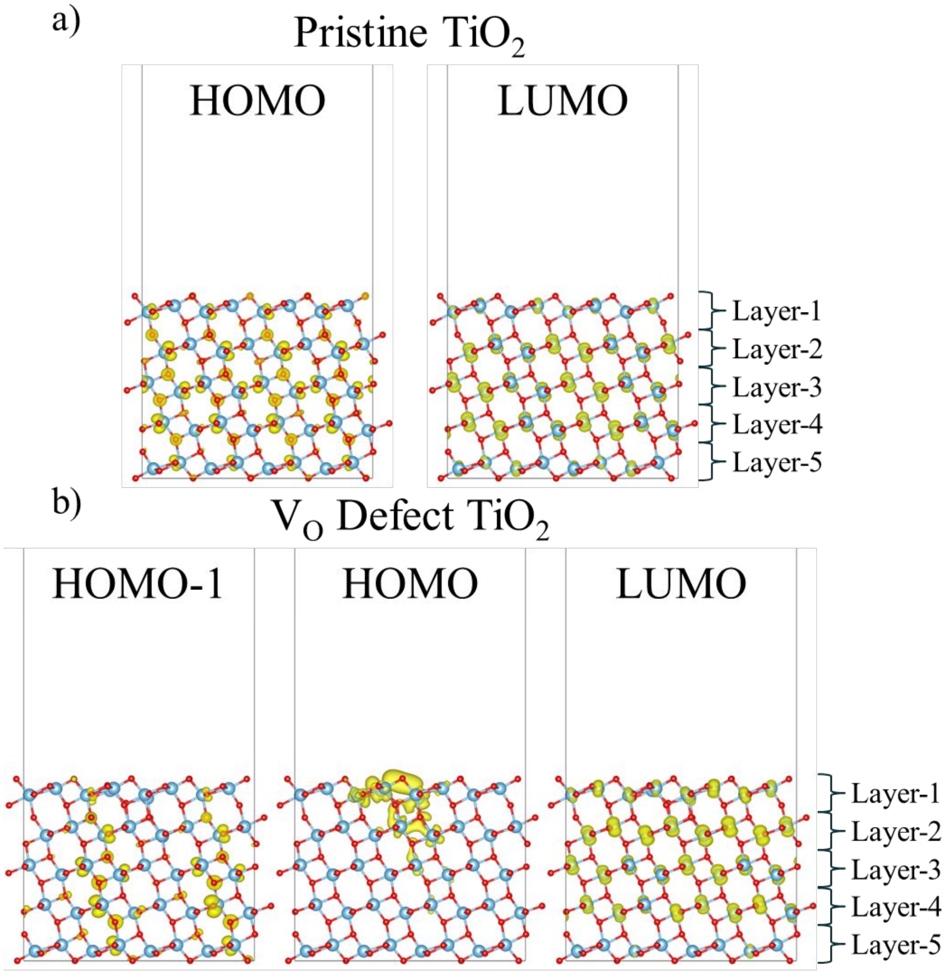

Figure S6.1. The partial (band-decomposed) charge density of the relevant band edge states (HOMO-1. HOMO, LUMO) at the Γ-point and 0 K for a) Pristine TiO_2 (101) and b) the V_O Defect TiO_2 slab models. In the pristine model the HOMO state is delocalized across O atoms and all layers (left), while the LUMO is delocalized across Ti atoms and all layers (right). In the defect model, the HOMO-1 state is delocalized across O atoms and all layers (left). The HOMO state is a trap state and is localized primarily within the top two layers near the oxygen vacancy site (middle). The LUMO state is delocalized across the Ti atoms within the top 4 layers (layers 1-4) (right).

S7: Planar-averaged Electrostatic Potential for Pristine and V_O Defect TiO_2 Slab

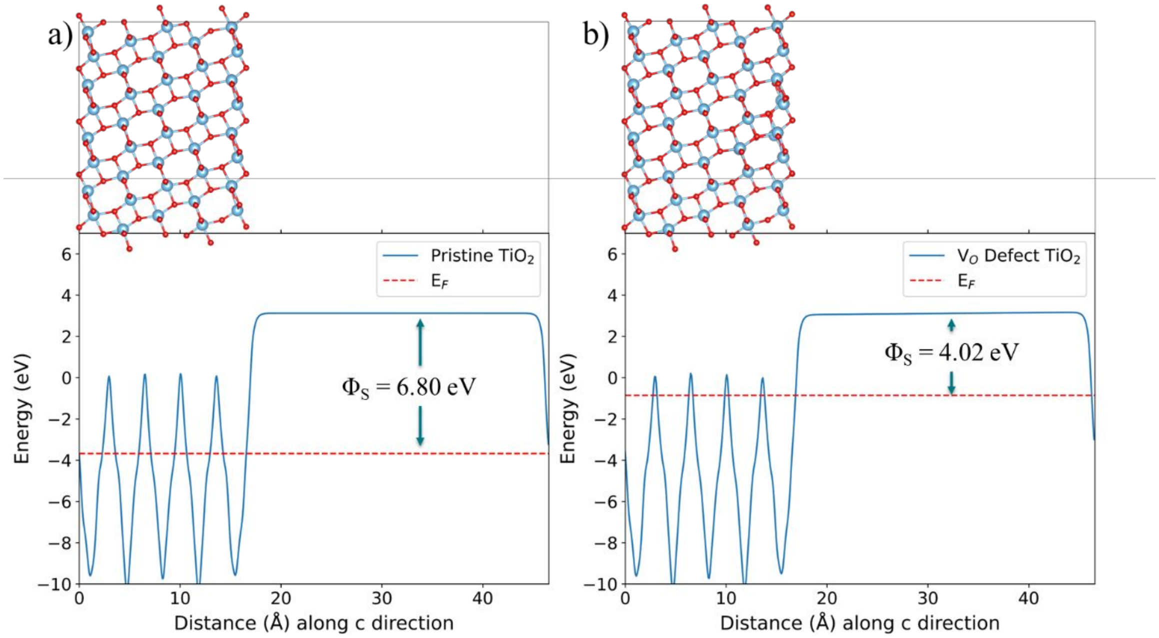

Figure S7.1. Planar-average electrostatic potential along the c direction at 0 K, Γ-point for a) Pristine TiO_2 and b) the V_O Defect TiO_2 slab models. The flat region of the potential (blue line) represents the vacuum potential ϕ_{vacuum}, and the red dashed line represents E_F. The structure is overlaid to highlight atomic positions and the vacuum region. The difference between ϕ_{vacuum} and E_F signifies the Φ of the surface. Note that the pristine Φ value (6.80 eV) differs slightly from that in **Figure S1.2d** (6.82 eV) due to differences in K-point grid sampling.

For semiconducting materials, E_F is not uniquely defined in the bandgap in VASP calculations; any energy within the gap that satisfies the electron-count condition is acceptable.[12] Typically, VASP positions E_F near the bottom of the bandgap, but the exact placement depends on the smearing and the density of states parameters. In the V_O defect TiO_2 system, the formation of an additional occupied state (HOMO in **Figure 1d**) at significantly higher energy compared to the pristine HOMO (**Figure 1c**) raises E_F, thereby lowering the work function. Because this filled defect state inherently positions E_F higher in energy than in the pristine TiO_2 system, comparisons of work function trends between pristine and V_O defect TiO_2 surfaces remain valid.

S8: Contribution Decomposed Eigen Band Energies for AuNPs–TiO_2 heterojunctions

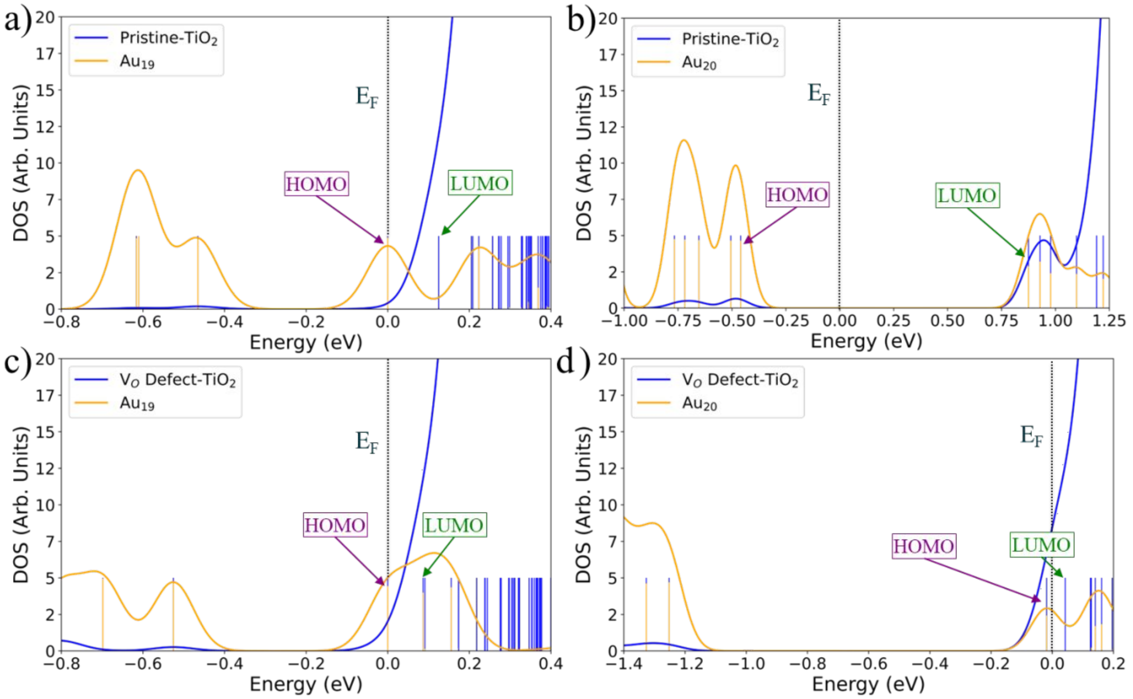

Figure S8.1. DOS plots of the AuNPs–TiO_2 heterojunctions, with contribution decomposed eigen band energies as sticks, evaluated at the Γ-point and 0 K. The E_F is set to zero. The TiO_2 contributions are blue, and the AuNPs contributions are orange. a) Au_{19}NP and pristine-TiO_2, b) Au_{20}NP–pristine-TiO_2, c) Au_{19}NP–V_O defect TiO_2, and d) Au_{20}NP–V_O defect TiO_2 heterojunctions.

S9: DOS Plots for Au_{19}NP and Au_{20}NP

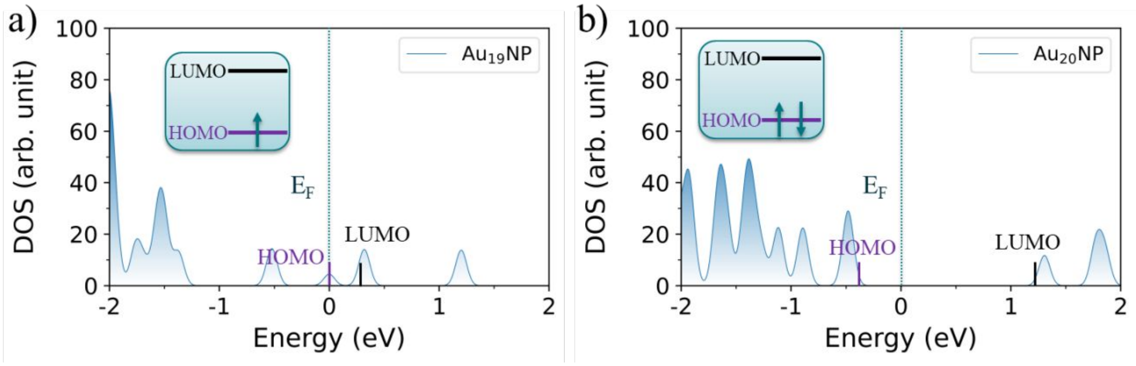

Figure S9.1. DOS plots at 0 K and the Γ-point for the isolated a) the Au_{19}NP and b) Au_{20}NP. The E_F is set to zero for all DOS plots. The electron occupancy at the HOMO (purple) and LUMO (black) levels are also shown as inserts.

S10: Near-degeneracy States for Au_{19}NP–V_O Defect TiO_2

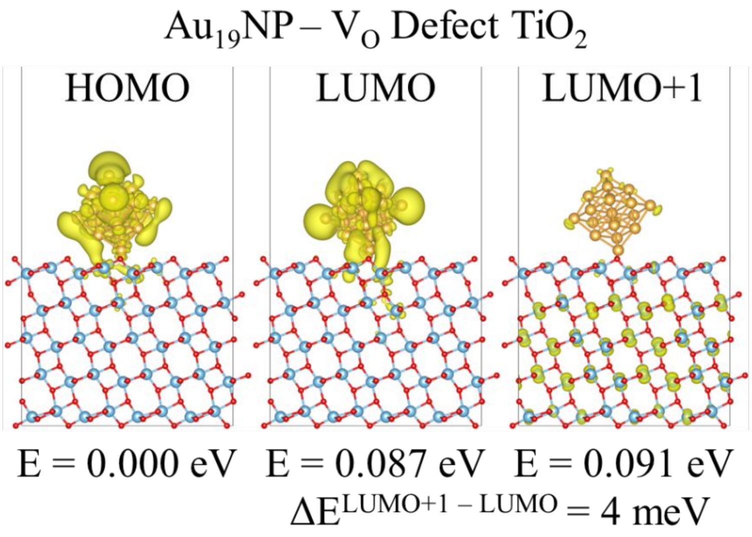

Figure S10.1. The band-decomposed charge density plots at the HOMO, LUMO, and LUMO+1 levels for Au_{19}NP–V_O defect TiO_2 at the Γ-point and 0 K. The band energy is shown below each plot, where the HOMO energy is set to zero. The LUMO and LUMO+1 levels are nearly degenerate, with a difference in energy of only 4 meV.

S11: Au DOS Contributions for AuNPs–TiO_2 heterojunctions

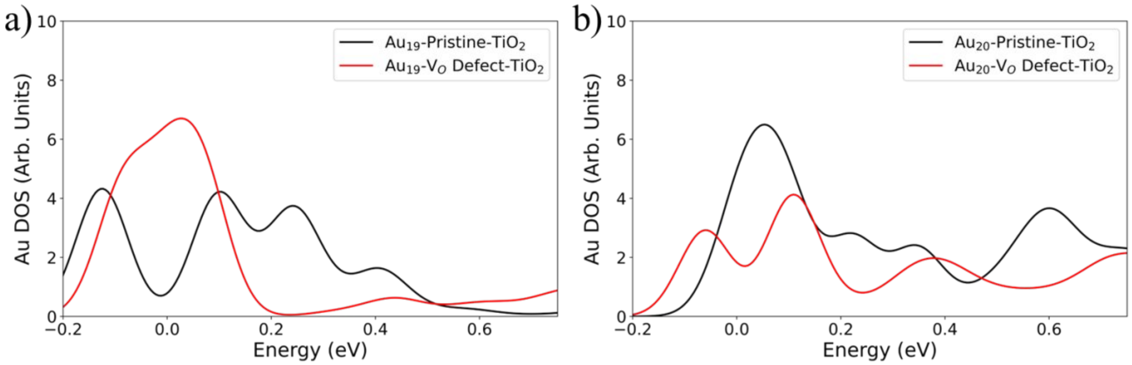

Figure S11.1. Au DOS contributions for a) the Au_{19}NP on a pristine TiO_2 surface (black) and an oxygen vacancy TiO_2 surface (red) at 0 K and the Γ-point. The LUMO is set to zero for both plots. A higher density of low-energy Au-associated CB states between 0-0.1 eV is observed for the defect surface. b) the Au_{20}NP on a pristine TiO_2 surface (black) and an oxygen vacancy TiO_2 surface (red) at 0 K and the Γ-point. A higher density of low-energy Au-associated CB states between 0-0.1 eV is observed for the pristine surface. This DOS comparison indicates that the TiO_2 surface plays a role in the electronic organization of states in Au.

S12: Bader Charge Analysis of the DFT Optimized Structures

Table S12.1. Layer resolved Bader charges for the pristine and V_O defect TiO_2 surface slabs systems at 0 K (structures are shown in **Figure 1**).

Bader Charge (*e*)	Pristine TiO_2	V_O defect TiO_2
**Layer 1**	0.18	0.56
**Layer 2**	-0.08	-0.34
**Layer 3**	-0.16	-0.25
**Layer 4**	-0.13	-0.14
**Layer 5**	0.19	0.18

Table S12.2. Layer resolved Bader charges for all AuNP–TiO_2 heterojunction systems at 0 K (structures are shown in **Figure S5.1**).

Bader Charge (*e*)	Au_{19}NP Pristine TiO_2	Au_{19}NP V_O defect TiO_2	Au_{20}NP Pristine TiO_2	Au_{20}NP V_O defect TiO_2
**Au**	-0.02	-0.64	0.17	-0.26
**Layer 1**	0.19	0.85	-0.02	0.63
**Layer 2**	-0.15	-0.16	-0.10	-0.20
**Layer 3**	-0.18	-0.18	-0.17	-0.23
**Layer 4**	-0.06	-0.08	-0.08	-0.13
**Layer 5**	0.21	0.22	0.20	0.19

S13: Molecular Dynamics of AuNP–TiO_2 Heterojunction

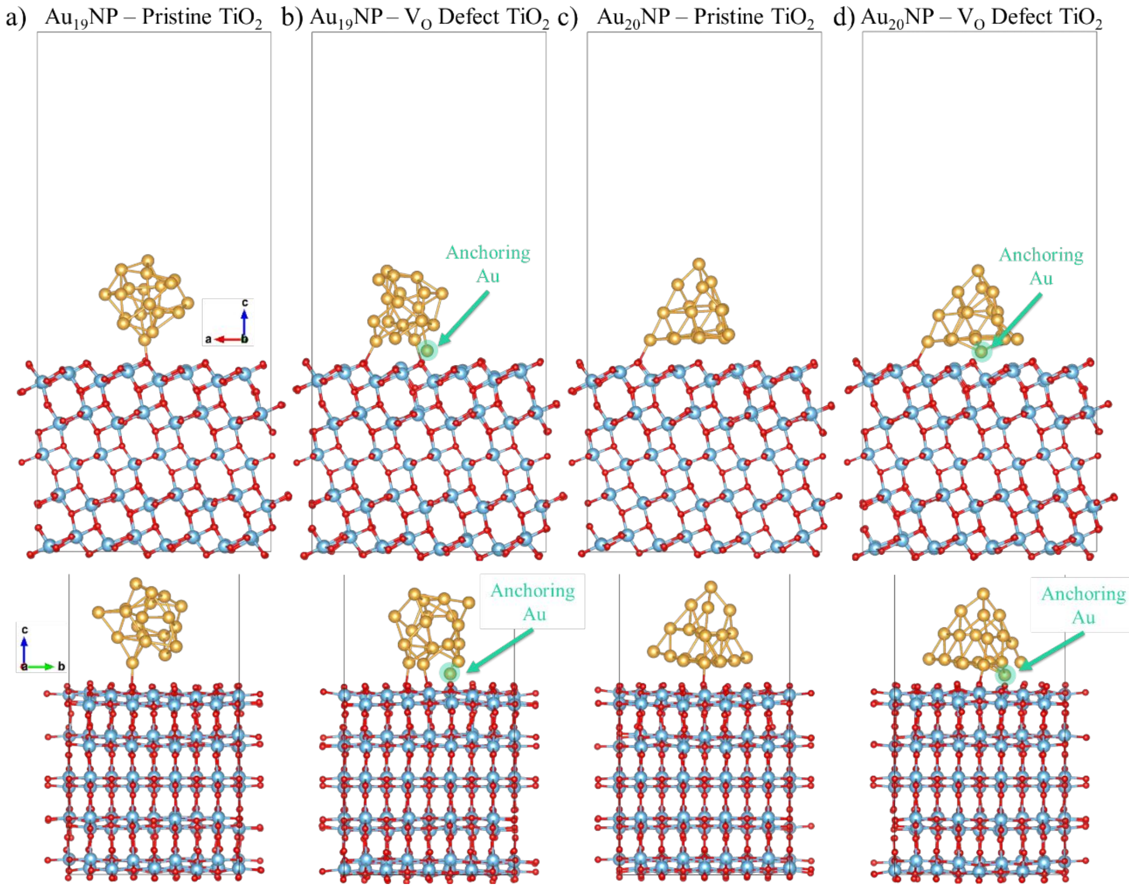

Figure S13.1. Representative structure models along the 6 ps trajectory viewed down the b-axis (top) and a-axis (bottom) for a) the Au_{19} Wulff constructed NP on the pristine TiO_2 surface, b) the Au_{19}NP on the V_O Defect TiO_2 surface, c) the Au_{20} "magic" NP on the pristine TiO_2 surface, and d) the Au_{20}NP on the V_O Defect TiO_2 surface at 300 K. The Au atom filling the vacancy site and anchoring the AuNP during the AIMD trajectory is highlighted in green.

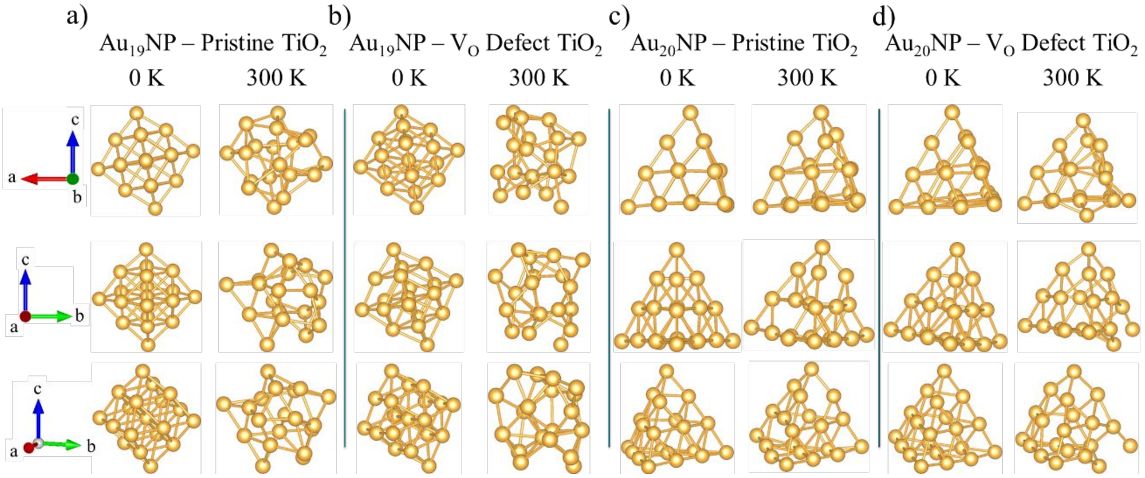

Figure S13.2. Structural comparison of the AuNP at 0 K and 300 K, viewed along the b-axis (top), a-axis (middle), and angled (bottom) for a) the Au_{19} Wulff constructed NP on the pristine TiO_2 surface, b) the Au_{19}NP on the V_O Defect TiO_2 surface, c) the Au_{20} "magic" NP on the pristine TiO_2 surface, and d) the Au_{20}NP on the V_O Defect TiO_2 surface.

Table S13.1. The HOMO–LUMO gap of the AuNP–TiO_2 heterojunctions at snapshots 1, 1500, 3000, 4500, 6000 fs, the total average along the 6 ps 300 K NVE trajectory, and the DFT optimized geometry at 0 K, Γ-point denoted "Static". Characteristic variations along the trajectories are quantified by standard deviations, stated together with the 6 ps average values.

**HOMO–LUMO Gap (eV)**	**Au_{19}NP Pristine TiO_2**	**Au_{19}NP V_O defect TiO_2**	**Au_{20}NP Pristine TiO_2**	**Au_{20}NP V_O defect TiO_2**
**1 fs**	0.21	0.20	1.06	0.73
**1500 fs**	0.47	0.23	1.07	0.89
**3000 fs**	0.17	0.18	0.93	0.88
**4500 fs**	0.21	0.30	0.72	0.68
**6000 fs**	0.29	0.25	1.15	0.77
**6 ps Average**	0.29±0.10	0.27±0.12	1.04±0.11	0.92±0.10
**Static**	0.12	0.09	1.33	0.06

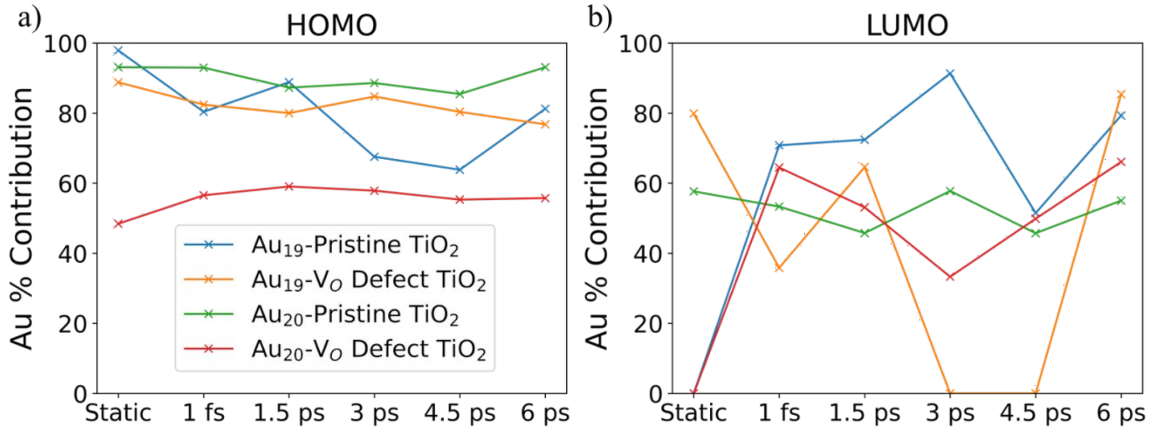

Figure S13.3. The AuNP percentage contribution of all heterojunctions to the a) HOMO and b) LUMO levels at the DFT optimized geometry denoted "Static" and several MD snapshots geometries (1, 1500, 3000, 4500, 6000 fs) along the 6 ps NVE trajectory. In all cases the HOMO is dominated by the AuNP (>50%), while the LUMO is much more dynamic in its Au character.

Table S13.2. The HOMO-1–LUMO gap of the AuNP–TiO_2 heterojunctions at snapshots 1, 1500, 3000, 4500, 6000 fs, the total average along the 6 ps 300 K NVE trajectory, and the DFT optimized geometry at 0 K, gamma point denoted "Static".

HOMO-1–LUMO Gap (eV)	Au_{19}NP Pristine TiO_2	Au_{19}NP V_O defect TiO_2	Au_{20}NP Pristine TiO_2	Au_{20}NP V_O defect TiO_2
**1 fs**	0.87	0.79	1.11	1.10
**1500 fs**	0.90	0.97	1.20	1.14
**3000 fs**	0.80	0.73	1.19	1.18
**4500 fs**	0.99	1.09	0.94	1.15
**6000 fs**	1.06	1.00	1.34	1.29
**6 ps Average**	0.98±0.12	0.94±0.13	1.16±0.10	1.16±0.10
**Static**	0.57	0.59	1.38	1.26

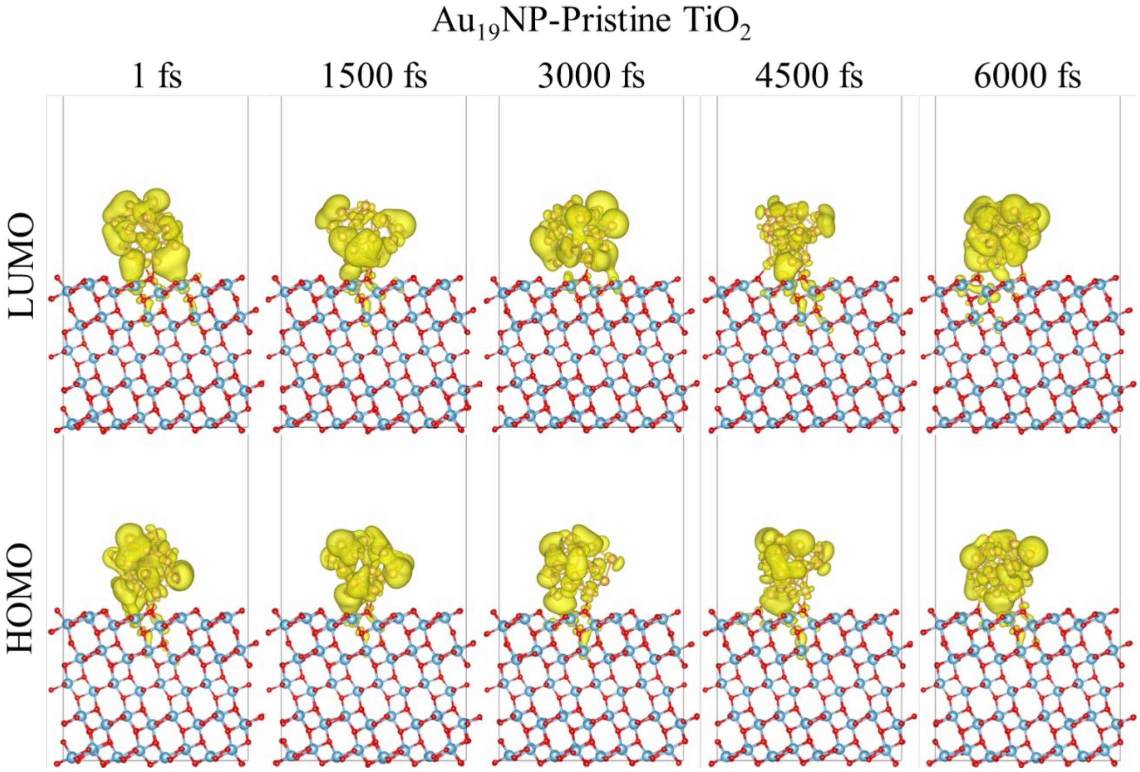

Figure S13.4. The partial (band-decomposed) charge density for several snapshots (1, 1500, 3000, 4500, 6000 fs) at the HOMO and LUMO levels along the 6 ps MD NVE trajectory of the Au_{19}NP–Pristine TiO_2 heterojunction at the Γ-point and 300 K. Comparing to the static Au_{19}NP–Pristine TiO_2 picture (**Figure 3a**), all LUMO changed from TiO_2 localization in the static picture (**Figure 3a**) to predominantly Au localization in this dynamical picture (**Figure S13.3**).

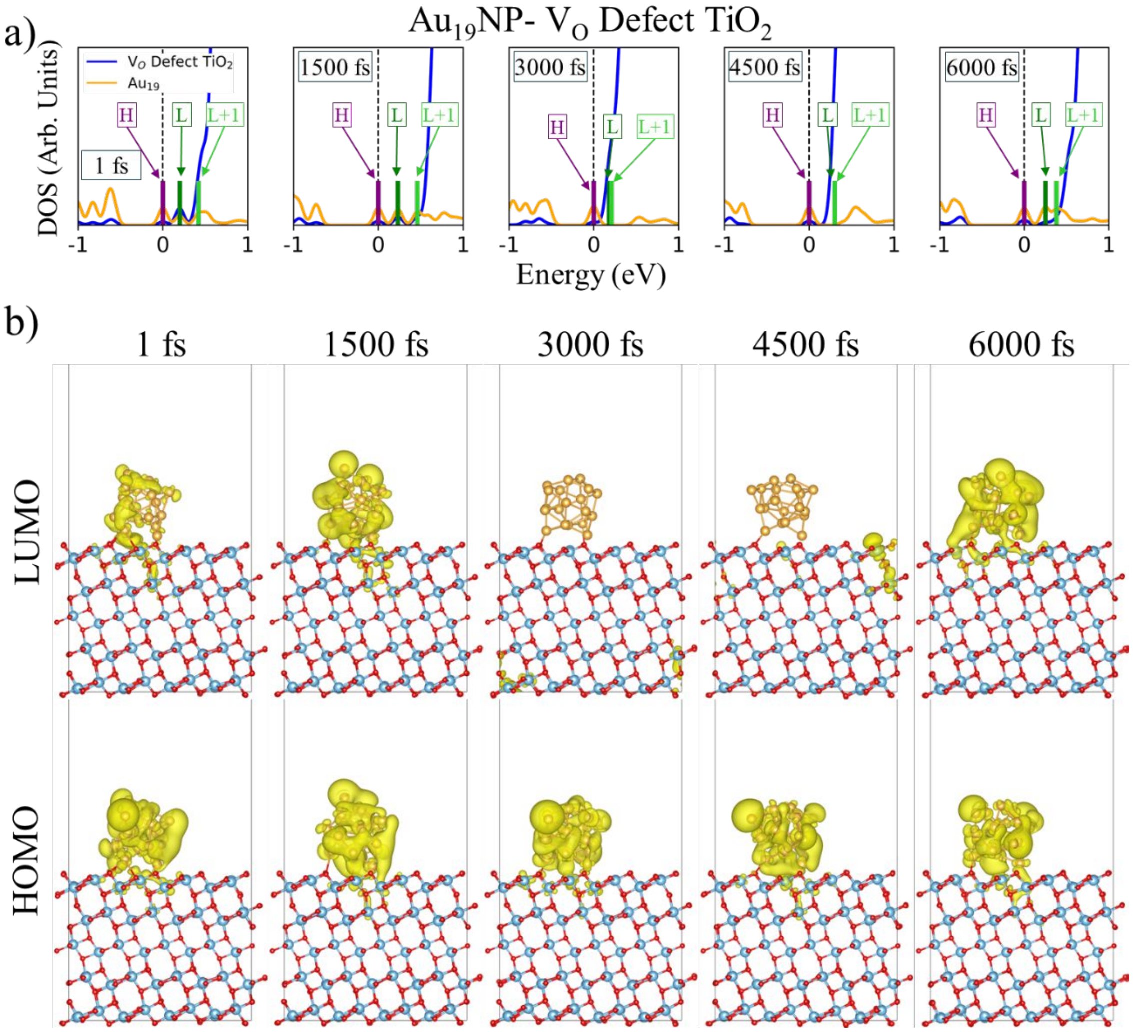

Figure S13.5. a) Calculated DOS for the Au_{19}NP–V_O defect TiO_2 heterojunction at time 1 fs, 1500 fs, 3000 fs, 4500 fs, and 6000 fs. The HOMO is shown in purple with label "H", the LUMO is shown in green with label "L", and the LUMO+1 is shown in lime green with label "L+1". At 3000 fs and 4500 fs, the LUMO and LUMO+1 are energetically degenerate, consistent with observations for the 0 K structure (**Figure S10.1**). b) The partial (band-decomposed) charge density for several snapshots (1, 1500, 3000, 4500, 6000 fs) at the HOMO and LUMO levels along the 6 ps MD NVE trajectory of the Au_{19}NP–V_O defect TiO_2 heterojunction at the Γ-point and 300 K. Similar to the static Au_{19}NP–V_O defect TiO_2 picture (**Figure 3b**), most HOMO and LUMO plots remain primarily localized on the AuNP in this dynamical picture (**Figure S13.4**), when the LUMO and LUMO+1 are not near energetic degeneracy (Step 1, 1500, 6000fs). However, when the LUMO and LUMO+1 are near degeneracy (ΔE <5meV), as in steps 3000 and 4500 fs, the LUMO becomes localized on the TiO_2 slab surface (layer 1 or 5, recall **Figure 1** for layer locations).

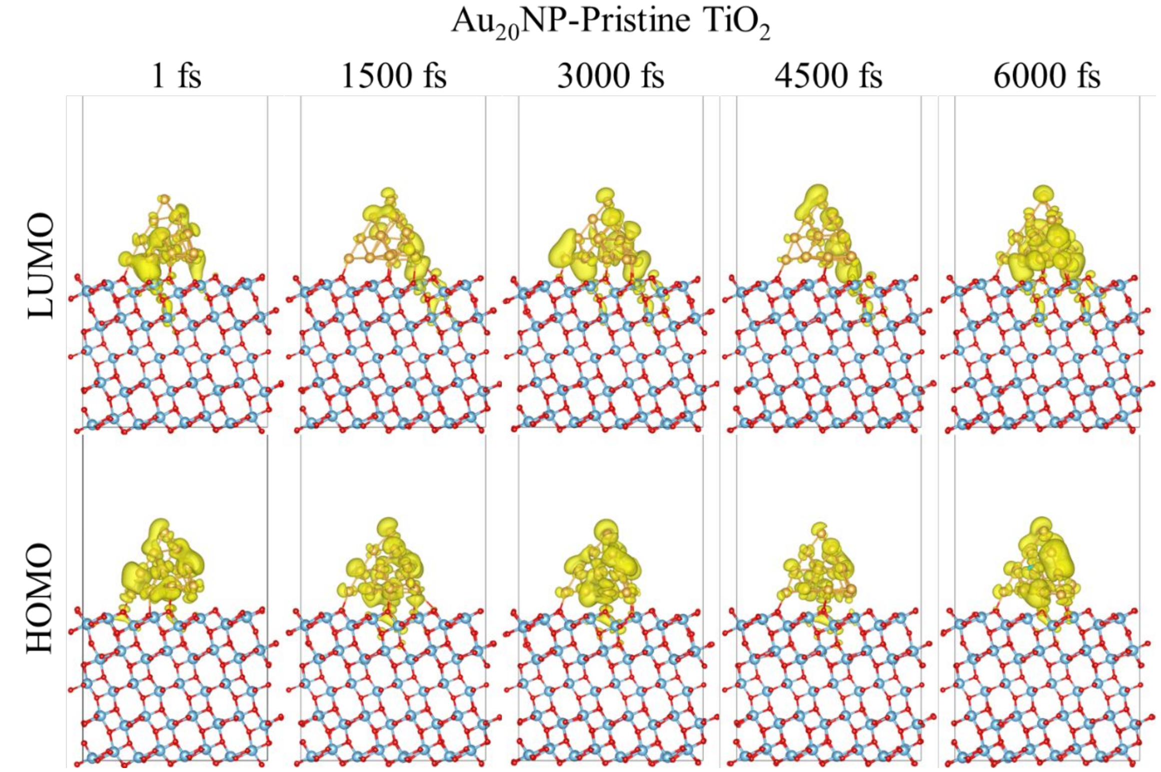

Figure S13.6. The partial (band-decomposed) charge density for several snapshots (1, 1500, 3000, 4500, 6000 fs) at the HOMO and LUMO levels along the 6 ps MD NVE trajectory of the Au_{20}NP–Pristine TiO_2 heterojunction at the Γ-point and 300 K. Similar to the static Au_{20}NP–Pristine TiO_2 picture (**Figure 3c**), all HOMO and LUMO plots remain primarily localized on the AuNP in this dynamical picture (**Figure S13.5**).

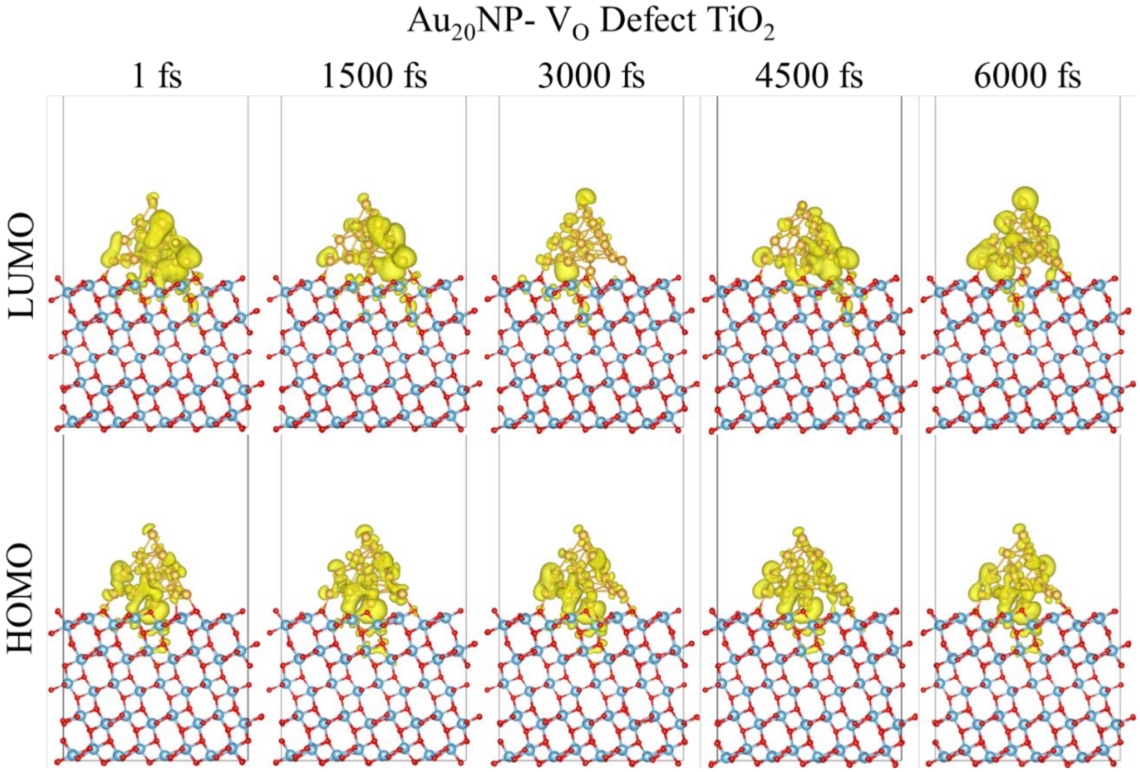

Figure S13.7. The partial (band-decomposed) charge density for several snapshots (1, 1500, 3000, 4500, 6000 fs) at the HOMO and LUMO levels along the 6 ps MD NVE trajectory of the Au_{20}NP–V_O defect TiO_2 heterojunction at the Γ-point and 300 K. Comparing to the static Au_{20}NP–V_O defect TiO_2 picture (**Figure 3d**), all LUMO changed from TiO_2 localization in the static picture (**Figure 3d**) to predominantly Au localization in this dynamical picture (**Figure S13.6**).

S14: Bader Charge Analysis of the AIMD Structures

Table S14.1. Bader charges for all four heterojunctions at elevated temperatures. Bader charges for the AuNP and TiO_2 layers are averaged over five 300 K AIMD snapshots (1, 1500, 3000, 4500, 6000 fs) and compared to the static 0 K results.

Bader Charge (*e*)	Au_{19}NP Pristine TiO_2		Au_{19}NP V_O defect TiO_2		Au_{20}NP Pristine TiO_2		Au_{20}NP V_O defect TiO_2	
	**Static**	**AIMD**	**Static**	**AIMD**	**Static**	**AIMD**	**Static**	**AIMD**
**AuNP**	-0.02	0.15 ± 0.04	-0.64	-0.50 ± 0.05	0.17	0.22 ± 0.03	-0.26	-0.43 ± 0.04
**Layer 1-TiO_2**	0.19	0.00 ± 0.04	0.85	0.62 ± 0.04	-0.02	-0.14 ± 0.06	0.63	0.55 ± 0.10
**Layer 2-TiO_2**	-0.15	-0.19 ± 0.04	-0.16	-0.21 ± 0.03	-0.10	-0.15 ± 0.10	-0.20	-0.15 ± 0.05
**Layer 3-TiO_2**	-0.18	0.11±0.05	-0.18	0.13±0.04	-0.17	0.13±0.12	-0.23	0.10±0.05
**Layer 4-TiO_2**	-0.06	-0.21±0.08	-0.08	-0.16±0.04	-0.08	-0.17±0.05	-0.13	-0.19±0.04
**Layer 5-TiO_2**	0.21	0.15±0.07	0.22	0.12±0.02	0.20	0.11±0.05	0.19	0.12±0.03

Table S14.2. Layer resolved Bader charges for the Au_{19}NP–Pristine TiO_2 heterojunction for several AIMD 300 K snapshots (1, 1500, 3000, 4500, 6000 fs), the 0 K "static" Bader charges are reproduced for comparison.

**Au_{19}NP–Pristine TiO_2**							
**Units (*e*)**	**Static**	**1 fs**	**1500 fs**	**3000 fs**	**4500 fs**	**6000 fs**	**Snapshot Average**
**AuNP**	-0.02	0.10	0.10	0.18	0.18	0.17	0.15±0.04
**Layer 1-TiO_2**	0.19	0.04	0.03	-0.02	-0.01	-0.06	0.00±0.04
**Layer 2-TiO_2**	-0.15	-0.22	-0.19	-0.16	-0.23	-0.15	-0.19±0.04
**Layer 3-TiO_2**	-0.18	0.17	0.07	0.08	0.16	0.06	0.11±0.05
**Layer 4-TiO_2**	-0.06	-0.19	-0.12	-0.18	-0.32	-0.24	-0.21±0.08
**Layer 5-TiO_2**	0.21	0.10	0.10	0.09	0.22	0.22	0.15±0.07

Table S14.3. Layer resolved Bader charges for the Au_{19}NP–V_O defect TiO_2 heterojunction for several AIMD 300 K snapshots (1, 1500, 3000, 4500, 6000 fs). The 0 K "static" Bader charges from **Table S12.2** are reproduced for comparison.

**Au_{19}NP–V_O defect TiO_2**							
**Units (*e*)**	**Static**	**1 fs**	**1500 fs**	**3000 fs**	**4500 fs**	**6000 fs**	**Snapshot Average**
**AuNP**	-0.64	-0.48	-0.47	-0.56	-0.52	-0.44	-0.50±0.05
**Layer 1-TiO_2**	0.85	0.60	0.62	0.68	0.57	0.61	0.62±0.04
**Layer 2-TiO_2**	-0.16	-0.18	-0.21	-0.23	-0.18	-0.26	-0.21±0.03
**Layer 3-TiO_2**	-0.18	0.07	0.10	0.15	0.15	0.17	0.13±0.04
**Layer 4-TiO_2**	-0.08	-0.15	-0.15	-0.19	-0.11	-0.21	-0.16±0.04
**Layer 5-TiO_2**	0.22	0.14	0.11	0.14	0.10	0.12	0.12±0.02

Table S14.4. Layer resolved Bader charges for the Au_{20}NP–Pristine TiO_2 heterojunction for several AIMD 300 K snapshots (1, 1500, 3000, 4500, 6000 fs). The 0 K "static" Bader charges from **Table S12.2** are reproduced for comparison.

**Au_{20}NP–Pristine TiO_2**							
**Units (*e*)**	**Static**	**1 fs**	**1500 fs**	**3000 fs**	**4500 fs**	**6000 fs**	**Snapshot Average**
**AuNP**	0.17	0.23	0.21	0.26	0.19	0.20	0.22±0.03
**Layer 1-TiO_2**	-0.02	-0.10	-0.18	-0.20	-0.18	-0.05	-0.14±0.06
**Layer 2-TiO_2**	-0.10	-0.17	-0.06	-0.19	-0.03	-0.28	-0.15±0.10
**Layer 3-TiO_2**	-0.17	0.06	0.05	0.13	0.10	0.34	0.13±0.12
**Layer 4-TiO_2**	-0.08	-0.16	-0.16	-0.11	-0.21	-0.23	-0.17±0.05
**Layer 5-TiO_2**	0.20	0.14	0.14	0.11	0.14	0.03	0.11±0.05

Table S14.5. Layer resolved Bader charges for the $Au_{20}NP–V_O$ defect TiO_2 heterojunction for several AIMD 300 K snapshots (1, 1500, 3000, 4500, 6000 fs). The 0 K "static" Bader charges from **Table S12.2** are reproduced for comparison.

**$Au_{20}NP–V_O$ defect TiO_2**							
**Units (*e*)**	**Static**	**1 fs**	**1500 fs**	**3000 fs**	**4500 fs**	**6000 fs**	**Snapshot Average**
**AuNP**	-0.26	-0.41	-0.47	-0.48	-0.43	-0.39	-0.43±0.04
**Layer 1-TiO_2**	0.63	0.47	0.67	0.64	0.52	0.44	0.55±0.10
**Layer 2-TiO_2**	-0.20	-0.14	-0.22	-0.14	-0.16	-0.09	-0.15±0.05
**Layer 3-TiO_2**	-0.23	0.16	0.08	0.03	0.11	0.10	0.10±0.05
**Layer 4-TiO_2**	-0.13	-0.22	-0.22	-0.13	-0.20	-0.18	-0.19±0.04
**Layer 5-TiO_2**	0.19	0.13	0.15	0.08	0.15	0.10	0.12±0.03

S15: References

(1) Long, R.; Prezhdo, O. V. Instantaneous Generation of Charge-Separated State on TiO2 Surface Sensitized with Plasmonic Nanoparticles. *J. Am. Chem. Soc.* **2014**, *136* (11), 4343–4354. https://doi.org/10.1021/ja5001592.

(2) Alexopoulos, K.; Hejduk, P.; Witko, M.; Reyniers, M.-F.; Marin, G. B. Theoretical Study of the Effect of (001) TiO2 Anatase Support on V2O5. *J. Phys. Chem. C* **2010**, *114* (7), 3115–3130. https://doi.org/10.1021/jp910685z.

(3) Martsinovich, N.; Jones, D. R.; Troisi, A. Electronic Structure of TiO2 Surfaces and Effect of Molecular Adsorbates Using Different DFT Implementations. *J. Phys. Chem. C* **2010**, *114* (51), 22659–22670. https://doi.org/10.1021/jp109756g.

(4) Vittadini, A.; Selloni, A.; Rotzinger, F. P.; Grätzel, M. Formic Acid Adsorption on Dry and Hydrated TiO2 Anatase (101) Surfaces by DFT Calculations. *J. Phys. Chem. B* **2000**, *104* (6), 1300–1306. https://doi.org/10.1021/jp993583b.

(5) Hammes-Schiffer, S.; Tully, J. C. Proton Transfer in Solution: Molecular Dynamics with Quantum Transitions. *J. Chem. Phys.* **1994**, *101* (6), 4657–4667. https://doi.org/10.1063/1.467455.

(6) Hu, P.; Lei, M.; Sui, Z.-J.; Zhou, X.-G.; Chen, D.; Zhu, Y.-A. Identifying the Active Phase on Atomically Dispersed Catalysts for Propane Dehydrogenation: Positively Charged vs Metallic Transition Metals. *ACS Catal.* **2024**, *14* (11), 8602–8618. https://doi.org/10.1021/acscatal.4c01372.

(7) Tang, H.; Prasad, K.; Sanjinès, R.; Schmid, P. E.; Lévy, F. Electrical and Optical Properties of TiO2 Anatase Thin Films. *J. Appl. Phys.* **1994**, *75* (4), 2042–2047. https://doi.org/10.1063/1.356306.

(8) Barmparis, G. D.; Lodziana, Z.; Lopez, N.; Remediakis, I. N. Nanoparticle Shapes by Using Wulff Constructions and First-Principles Calculations. *Beilstein J. Nanotechnol.* **2015**, *6*, 361–368. https://doi.org/10.3762/bjnano.6.35.

(9) Kryachko, E. S.; Remacle, F. The Magic Gold Cluster Au20. *Int. J. Quantum Chem.* **2007**, *107* (14), 2922–2934. https://doi.org/10.1002/qua.21504.

(10) Li, J.; Li, X.; Zhai, H.-J.; Wang, L.-S. Au20: A Tetrahedral Cluster. *Science* **2003**, *299* (5608), 864–867. https://doi.org/10.1126/science.1079879.

(11) Tarrat, N.; Rapacioli, M.; Cuny, J.; Morillo, J.; Heully, J.-L.; Spiegelman, F. Global Optimization of Neutral and Charged 20- and 55-Atom Silver and Gold Clusters at the DFTB Level. *Comput. Theor. Chem.* **2017**, *1107*, 102–114. https://doi.org/10.1016/j.comptc.2017.01.022.

(12) *EFERMI - VASP Wiki*. https://www.vasp.at/wiki/index.php/EFERMI (accessed 2025-05-06).
